# Virome Survey of Banana Plantations and Surrounding Plants in Malawi

**DOI:** 10.3390/v17081068

**Published:** 2025-07-31

**Authors:** Johnny Isaac Gregorio Masangwa, Coline Temple, Johan Rollin, François Maclot, Serkan Önder, Jamestone Kamwendo, Elizabeth Mwafongo, Philemon Moses, Isaac Fandika, Sebastien Massart

**Affiliations:** 1 Plant Pathology Laboratory, Terra, Gembloux Agro-Bio Tech, University of Liege, Passage des Déportés, 2-5030 Gembloux, Belgiumsebastien.massart@uliege.be (S.M.); 2 Department of Agricultural Research Services, Bvumbwe Agricultural Research Station, Limbe P.O Box 5748, Malawi; 3 Department of Plant Protection, Faculty of Agriculture, Eskişehir Osmangazi University, Eskişehir 26160, Türkiye; 4 National Herbarium and Botanic Gardens of Malawi, Zomba P.O Box 528, Malawi; 5 Center for Health, Agriculture and Development Research and Consulting, Blantyre P.O Box 399, Malawi; 6 Department of Agricultural Research Services, Kasinthula Agricultural Research Station, Chikwawa P.O Box 28, Malawi

**Keywords:** high throughput sequencing, virome survey, novel viruses, new host plant, plant viruses, detection

## Abstract

A virome survey of banana plantations and their surrounding plants was carried out at nation-wide level in Malawi using virion associated nucleic acids (VANA) high throughput sequencing (HTS) on pooled samples and appropriate alien controls. In total, 366 plants were sequenced, and 23 plant virus species were detected, three species on banana (275 plants) and 20 species in surrounding plants (91 plants). Two putative novel virus species; ginger tymo-like virus and pepper derived totivirus were detected and confirmed by RT-PCR on ginger and pepper. Nine known virus species and detected a host plant was identified for two of them. No viral exchange between banana and surrounding plants was observed. Results from the VANA protocol, applied to pooled banana samples, were compared with previous targeted PCR results obtained from individual banana samples. HTS test detected better BanMMV than IC-(RT)-PCR on individual samples (better inclusivity) but detected with much lower sensitivity BBTV and BSV species, often with less than 10 reads per sample. Detection of novel and known viruses and new host plants calls for strengthened sanitory and phytosanitory measures within and beyond banana production systems. Our research confirms that HTS sensitivity depends on sampling, pooling protocol and targeted virus species.

## 1. Introduction

Banana is a vital food crop in Malawi and other tropical countries and is cultivated for its fruits that are eaten as dessert fruit or cooked. This crop is grown throughout Malawi, but the main producing districts are Chitipa, Karonga, and Nkhata-Bay in the northern region, Nkhotakota in the central region, and Thyolo and Mulanje in the southern region [1]. Banana production in Malawi experienced a significant decline in early 2000 due to an outbreak of banana bunchy top disease (BBTD), caused by the banana bunchy top virus (BBTV), which wiped out and continues to destroy bananas in previously unaffected areas [1]. Efforts are underway to revive banana industry in Malawi by eradicating the BBTV-infected banana plants and fostering the introduction of BBTV-free planting material. However, the geographical expansion of BBTV and its re-emergence in the newly established plantation where all old banana mats were removed has been recently confirmed [2]. Recently, seven additional viruses, six banana streak virus (BSV) species (banana streak Cavendish virus, BSCAV; banana streak Gold finger virus, BSGFV; banana streak Lacatan virus, BSV-Lac; banana streak Imové virus, BSIMV; banana streak Mysore virus, BSMYV and banana streak Obino l’Ewai virus, BSOLV) and the banana mild mosaic virus (BanMMV), were detected for the first time in the country using targeted molecular detection tests on individual plant samples [3].

High Throughput Sequencing (HTS) complemented by bioinformatics analyses is an emerging standard technology for detecting known and new viruses, and for studying their population diversity, epidemiology, and evolution [4]. For instance, HTS-based profiling of plant virus genomes enabled the reconstruction of the history of potato virus V evolution and dissemination [5] or the nation-wide analysis of population structure of Uganda cassava brown streak virus (UCBSV) [6,7]. According to Kutnjak et al. [7], there is a drastic intensified use of HTS technologies to detect known and new plant viruses and characterize the plant virome in different plant species. It represents now the gold standard to identify viruses causing novel diseases and diseases of unknown etiology [8]. HTS also offers the opportunity to characterize plant viruses in individual or pooled plant samples, allowing large scale territory surveillance [6,9,10] including baseline survey for identifying potentially emergent viruses [11].

As the virus concentration can be very low in plants [12], laboratory protocols were developed to enrich the library in viral sequences and, therefore, the dataset in viral sequences [12]. Such enrichment protocol is also particularly interesting when pooling plants as it maintains an acceptable analytical sensitivity. Virion-associated nucleic acids (VANAs) is one of the most popular enrichment approaches as it has already reliably detected viruses from 85 different families, including DNA and RNA viruses [13]. Yet, VANA detected fewer virus species than dsRNA for samples with a very high virus richness [14].

Virus exchange, mostly mediated by vectors, can occur between crops and weeds in both senses [15,16,17], influenced by the host compatibility, as well as the proximity and density of crops and weeds [17]. Current publications provide contrasting results. For example, Ma et al. [16] detected similar viruses in tomato (*Solanum lycopersicum*) and neighboring European black nightshade (*Solanum nigrum*) while Maachi [18] reported that neighboring wild and cultivated cucurbits did not share viruses.

Transfers of viruses from annual crops to weeds at the end of cultural season are vital as primary inoculum for the disease outbreak in the following season [19,20] because weeds can act as off-season reservoir of viruses which spill over to crops during the cropping season. For banana viruses, recent studies [20,21] detected BBTV in *Biden pilosa*, elephant ear (*Caladium bicolor*), heliconia (*Heliconia aurantiaca*) [20,21]. Banana bract mosaic virus (BBrMV) was detected in Cardamom (*Elettaria cardamomum* Maton) [22] while BanMMV was recently detected in yam (*Diascorea* spp.) [23]. The detection of banana viruses in weeds [20,21,22] or the detection of cactus virus X (CVX) in banana [24] confirmed the virus exchanges between banana and surrounding plants. To complement a virome characterization survey of banana plantation, there was therefore an opportunity to investigate if other plants species surrounding banana cultivation in Malawi could host banana viruses. This research, therefore, presents a nation-wide virome exploration of banana plantations and surrounding plants in Malawi using HTS technologies.

## 2. Materials and Methods

### 2.1. Plant Sample Collection and Species Identification

**Figure 1:**
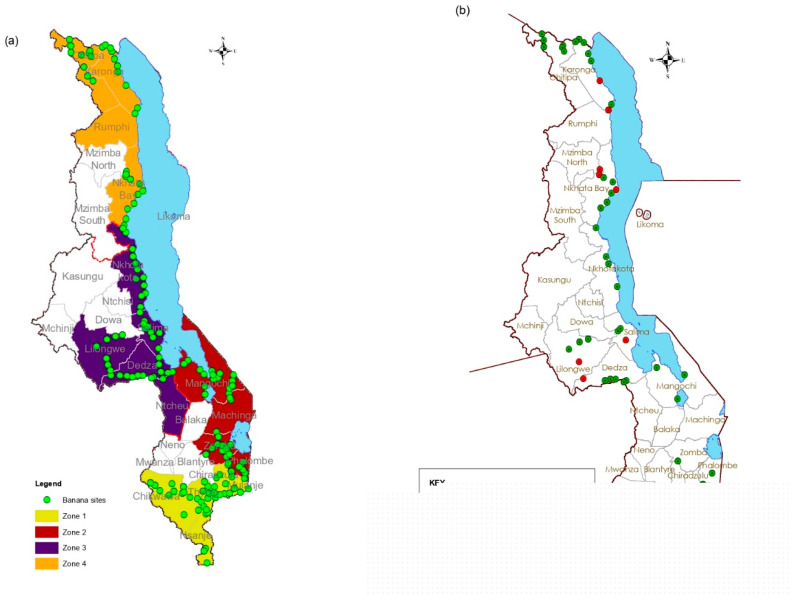
(**a**) Survey sites and banana cultivation zones. (**b**) Collection site for weeds and other plants. In (**a**) the green dots represent the survey sites and Zone 1 is shaded yellow, Zone 2 brown, Zone 3 dark blue and Zone 4 orange. For (**b**) green dots sites where samples of other crops were collected and red dots weed collection sites.

Plant samples were collected in four banana cultivation zones (see Figure 1a,b) described in a previous work [3]. In total, 366 individual plant samples, composed of 275 bananas and 91 other surrounding plants (belonging to 24 botanical families and 47 species) were collected in Malawi in 2020 (Table S1). The surrounding plants corresponded to crops (n = 74 samples from 31 species) and weeds (n = 17 samples from 16 species) that were growing adjacent to banana and plantains. The third youngest leaf or leaflet (depending on the leaf type) from the recently emerged leaves from the top of each plant was collected, wrapped in paper towels and put in individual zippable sample-collecting plastic bags. The samples collected were dried on silica gel. Georeferenced data of the survey sites; samples’ local and common names and their pictures (especially for weeds) were collected and entered on a survey form that was pre-uploaded on MDC-GIS Cloud software (v.8.1). Weed samples were identified at the National Herbarium and Botanical Gardens of Malawi. The collected leaf samples were then stored at −80 °C until use.

### 2.2. Sample Poolings Viral Enrichment and High Throughput Sequencing

**Figure 2:**
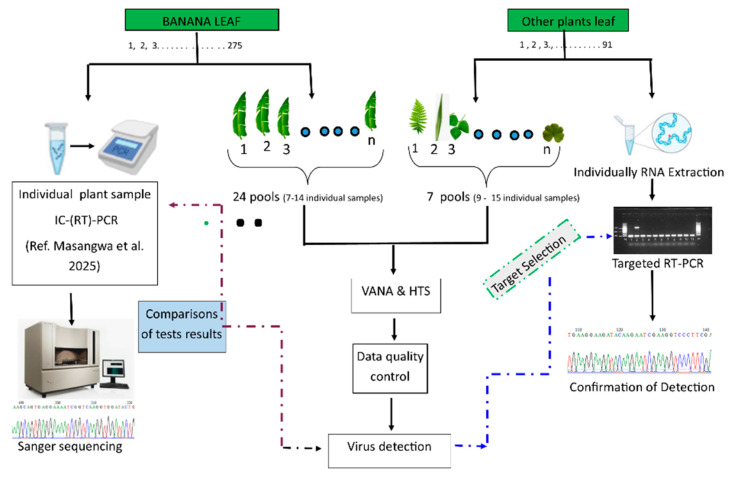
Schematic representation of sample pooling for VANA sequencing, the comparison of results with individual targeted test (IC-(RT)-PCR) from Masangwa et al. under review [3], and the independent confirmation of the new detections (new virus, new host and new to Malawi).

Plants were sampled irrespective of viral symptoms presence, however, plants with evident fungal infection or necrosis were discarded. Banana samples were individually tested for banana viruses using immunocapture reverse transcription polymerase chain reaction IC-(RT)-PCR as described in Masangwa et al. under review [3]. The banana samples were then pooled based on the district within banana cultivation zones (n = 21 asymptomatic pools) and three pools with symptomatic samples (1, 9, and 18) with 7 to 14 individual samples per pool as illustrated in Figure 2 and Table S2. To evaluate if neighboring plants can host banana viruses, samples from associated plants (crops and weeds) were pooled (n = 7 pools; 9–15 plants per pool) (Figure 2) based on closeness of the collection sites. The composition of the plant pools is detailed in Supplementary Tables S1 and S3.

Total nucleic acid extraction was carried out using a virion-associated nucleic acids enrichment protocol (VANA) before sequencing using Illumina technology, as described earlier [25]. VANA approach was selected as the best approach to be used in this study on the premise that it combines reverse transcriptase priming and Klenow treatment stage, allowing the reliable detection of both RNA and DNA viruses [13,26,27].

A mass-balanced proportion of each leaf sample was pooled in an extraction bag to sum up 10 g of starting plant material and stored at −80 °C [28]. Then, the 10 g of plant tissue were ground in cold Hanks’ buffered salt solution (HBSS) (50 mL), made up of 0.137 M NaCl, 5.4 mM KCl, 0.25 mM Na_2_HPO_4_, 0.07 g glucose, 0.44 mM KH_2_PO_4_, 1.3 mM CaCl_2_, 1.0 mM MgSO_4_, and 4.2 mM NaHCO_3_ using a motorized tissue homogenizer. The clarification was acquired from a centrifugation run of 10,000× *g* for 10 min at 4 °C in a 50 mL falcon tube. The supernatant was then filtered through a 0.45 μm sterile syringe filter.

Several controls were used according to the recommended guidelines for the use of HTS technologies in plant pest detection [29]. For evaluating the analytical sensitivity of the test for each pool, an alien internal control [30] was spiked in crude extract of each composite sample at 1:100 (*v*:*v*). This alien control corresponded to 370 µL of clarified bean (*Phaseolus vulgaris* L.) supernatant infected with three endornaviruses; Phaseolus vulgaris virus 1 (PvEV 1), 2 (PvEV 2), and 3 (PvEV 3), added in 37 mL of the filtered supernatant [29]. Tomato (*Solanum lycopersicum*) infected with Physostegia chlorotic mottle virus (PhCMoV; genus *Alphanucleorhabdovirus*) and orchid (*Cymbidium* spp.) infected with odontoglossum ringspot virus (ORSV, *Nepovirus*) were used as external alien controls while PCR water was used as a negative control during the amplifications.

A volume of 10.5 mL of spiked supernatants was transferred into ultracentrifuge tubes (Beckman Coulter ultra-clear 13.5-mL tubes (No. 344085); Beckman Coulter, Brea, CA, USA, to which 1 mL of 30% sucrose in 0.2 M potassium phosphate at pH 7.0 was added at the bottom of the tube to act as a sucrose cushion. Extracts were then centrifuged at 40,000 rpm for 2 h at 4 °C using the 50Ti rotor (Beckman Coulter). The pellets were resuspended in 1.5 mL of HBSS and stored overnight at 4 °C. From a 200 μL resuspension, unencapsidated nucleic acids were digested with 15 U of bovine pancreas DNase I (Euromedex, Souffelweyersheim, France) and 1.9 U of bovine pancreas RNase A (Euromedex, France) and incubated at 37 °C for 90 min. Encapsidated nucleic acids were then extracted using a PureLink Viral RNA/DNA Mini Kit (ThermoFischer Scientific, Waltham, MA, USA) following the manufacturer’s protocol [25].

The extracted nucleic acids from pools (35 in total = 31 samples + 4 controls) were then divided into 3 sets (2 sets of 12 samples each and 1 set with 11 pools with one external alien control for each set). The cDNA libraries were prepared as described [25] and twelve barcoded dodecamer (Dodec) primers were used to prepare the cDNAs. Each sample pool was assigned to a specific DoDec primers within the set (Table S3). This was followed by priming and extension of the cDNAs in Klenow Fragment DNA polymerase (Promega, Madison, WI, USA) program with the use of the same DoDec primers like in cDNA synthesis. The barcoded PCR products were mixed into the 3 libraries (Table S3), followed by a purification using Nucleospin Gel and PCR cleanup (Macherey-Nagel, Hoerdt, France). Libraries were further prepared using NEBNext Ultra II DNA library prep kit (New England BioLabs, Ipswich, MA, USA) at GIGA Genomics (University of Liege, Liège, Belgium) followed by sequencing on the Illumina NextSeq500 sequencer (San Diego, CA, USA) for the generation of 2 × 150 nt paired-end reads.

### 2.3. Bioinformatics Analyses

#### 2.3.1. HTS Data and Genomic Analyses

Bioinformatics analyses on HTS datasets were performed on Geneious Prime software (v.2022.2.1) (https://www.geneious.com, accessed on 4 August 2022) and Virhunter (v.2022.1.0.0) (https://github.com/virhunter/tree/main/virhunter, accessed on 22 September 2022) bioinformatics tools to detect the virus species present in the obtained sequences [31].

Within each library, the sequence reads were demultiplexed per pool using Cutadapt (v.4.0) and allowing 1 mismatch in Mobaxterm (v.12.4) according to the MIDs sequences. They were then trimmed from the adaptors, primers and low-quality bases with the use of BBDuk Adapter/Quality Trimming version 38.84 [30]. Afterwards, reads were paired and merged, followed by de novo assembly with RNA-SPAdes assembler version 3.15.5 using default parameters on Geneious Prime (v.2022.2.1) [32]. To back up this analysis, contigs were also assembled on Galaxy (https://assembly.usegalaxy.eu/, accessed on 6 August 2022) using RNAviral SPAdes (V3.15.5) with default parameters. All generated contigs were submitted to a tBlastx search on NCBI Viral RefSeq 214 database (downloaded in October 2022) with E-value threshold set at 10^−3^ using Geneious Prime to create a list of detected viruses for each dataset (corresponding to a pool of samples). The putative viral contigs were later confirmed by homology search using BLASTn against the NCBI nucleotide (nt) and BLASTx against NCBI non-redundant protein (nr) databases.

When required to retrieve nearly complete genome sequence, contig extension by iterative mapping (using Geneious mapper, custom sensitivity, none (fast/read mapping), 20% mismatch and maximum 3 nucleotides gaps allowed) was carried out in Geneious Prime (v2022.2). Amplification of the Vmethyltransf region, which had gap for one of the newly discovered virus, was carried out using new primers designed in IDT PrimerQuest^TM^ Tool (https://www.idtdna.com/pages/tools/primerquest, accessed on 11 September 2024) and the PCR product sequenced by sanger sequencing (Macrogen, Amsterdam, The Netherlands). For the novel virus species, the open reading frames (ORFs) and the functional gene domains were predicted by directly submitting the sequenced genomes on NCBI’s ORF finder (https://www.ncbi.nlm.nih.gov/orffinder/, accessed on 1 October 2022) and on conserved domain search tool (https://www.ncbi.nlm.nih.gov/Structure/cdd/wrpsb.cgi, accessed on 1 October 2022), respectively. The conserved domains were validated by HMMER v.3.3.2 (http://hmmer.org/, accessed on 9 October 2022).

#### 2.3.2. Pairwise Genome Comparison and Phylogenetic Analyses

The genome sequences of the viruses phylogenetically related to the new viruses were downloaded from the NCBI Genbank (Table S4). All genomes were then aligned using ClustalW (v.2.1) on Galaxy Europe (https://usegalaxy.eu/, accessed on 20 November 2024) with default parameters. After the alignment, ModelFinder was employed to identify the most suitable model for constructing the ML tree [33] and maximum likelihood phylogenetic trees were then constructed using IQ-TREE-Nucleotide (v.2.1.2) on the Galaxy Europe platform [34] with 1000 ultrafast bootstrap replicates [35].

Annotation of the phylogenetic tree was performed using Fig tree (v.1.4.4) (http://tree.bio.ed.ac.uk/software/figtree/, accessed on 22 November 2024). The amino acids sequences of the capsid protein (CP) and the RNA-dependent RNA polymerase (RdRp) for the newly discovered viruses were generated and compared to closely related species through pairwise alignment (as described above) and the construction of phylogenetic trees constructed using IQTREE Protein (v.2.1.2) on the Galaxy Europe platform in Galaxy with 1000 bootstraps replications. The pairwise identity matrices of the new genomes and proteins were compared to ICTV demarcation criteria (http://ictv.global, accessed on 23 November 2024) of the respective families to classify the detected viruses as known or novel viruses.

CABI digital library (https://www.cabidigitallibrary.org/journal/dmpd, accessed on 24 November 2024) and literature review were used to classify detected viruses as endemic to Malawi or newly detected in the country.

### 2.4. Confirmation of HTS-Based Virus Detection by (RT)-PCR

To reach the highest level of reliability, confirmation of the detected plant viruses was carried out in three cases: first-time detection in Malawi for nine known virus species, new host candidate of a known virus and discovery of novel viruses. The selected test was RT-PCR as it allows a rapid set up while presenting good analytical sensitivity [36]. The detection of viruses was carried out from the individual plant samples constituting the pool in which the virus was detected. Whenever possible, the amplified RT-PCR products were then purified and Sanger sequenced by Macrogen (Amsterdam, The Netherlands). The Sequences of the detected viruses were then deposited at NCBI.

#### 2.4.1. Total Nucleic Acid Extraction

Total RNA was extracted from all neighboring plants, corresponding to 91 individual plant samples belonging to 24 families and 49 species (Table S1). For each sample, 50 mg of leaf tissue from individual bag was weighed and then processed using the kit RNeasy^®^ Plus Mini Kit (Method S1 for the detailed protocol). RNA quality and concentration were determined by Nanodrop^®^ ND-1000 Spectrophotometer (ISOGEN Life Science, De Meern, The Netherlands).

#### 2.4.2. Reverse Transcription and PCR

The extracted and purified RNAs were reverse transcribed using the SuperScript III RT kit (Invitrogen, Carlsbad, CA, USA) and random hexamers (Invitrogen, Carlsbad, CA, USA) (Method S1 for the detailed protocol). The cDNAs were then tested for 11 virus species with existing primers from literature or newly designed (Table S5). PCR protocols for novel viruses were developed, tested, and optimized using gradient PCR. Briefly, two microliters of the synthesized cDNAs were amplified in a 25 µL total volume reaction and Mango Taq polymerase (Biotium, Fremont, CA, USA). For each virus, the PCR tests contents and conditions (i.e., primers, Ta, and amplicon length) are presented in Table S5.

PCR products were analyzed by electrophoresis on a 1% agarose gel in 1× TAE (Tris-acetate-EDTA) buffer (composed of 40 mM Tris base, 20 mM Acetic acid, 1 mM EDTA), stained with GelRed nucleic acid gel stain (Biotium, Fremont, CA, USA), and visualized under UV light.

The amplicons for the detected viruses were cut using a sterile scalpel under UV light and put in a 2 mL microcentrifuge tube. The purification of the amplicon was done using Macherey-Nagel DNA, RNA and Protein purification kit (Duren, Germany). According to the manufacturer’s instructions. The quality assessment of the purified amplicons was done using Thermo Scientific NanoDrop 2000 Spectrophotometer. The pair of primers (forward and reverse) for the detected viruses were prepared in separate microcentrifuge tubes by diluting them to 10 µM concentration. The sequencing was done by Macrogen-Europe (Amsterdam, The Netherlands).

## 3. Results

### 3.1. Plant Sample Collection and Identification

A total of 366 individual leaf samples were collected of 47 plant species and 27 families were collected (Table S1). Two hundred seventy-five (275) samples were of *Musa* family and 91 plants were of other families (26) found to be growing adjacent to banana mats. Only 13 out of 91 plants were found growing adjacent to banana mats were weeds, and 78 were field and tree crops of economic importance. *Araceae*, *Euphorbiaceae*, *Fabaceae*, and *Solanaceae* plant families collected adjacent to banana mats recorded the highest (10–13) samples compared to other families. Annual crops that were found to be commonly intercropped with bananas were *Colocasia esculenta* (taro) (10), *Manihot esculanta* (cassava) (10), *Saccharum officinarum* (sugarcane) (6), *Solanum lycopersicum* (tomato) (4), and *Ipomoea batatas* (sweet potato) (4).

### 3.2. Virome Characterization by VANA-HTS

The obtained sequences are available in Sequence Read Archives (SRA, bioproject number PRJNA1193526). The HTS banana pools contained between 24,746 and 1,282,160 reads, while pools made up of weeds and other crops had 63,884 to 1,238,820 reads (Table S6). The internal alien controls (PvEV 1, PvEV 2 and PvEV 3) were detected in all samples with a read number ranging between 1084 and 286,742. The two alien viruses PhCMoV and ORV were detected in the external alien controls (n = 3) with read numbers ranging between 7865 and 295,959. From 7 to 34 contaminating reads of ORV (first external control) were detected in five pools (1, 6, 17, 29, and 31), representing a maximum contamination rate of 0.01%. PhCMoV reads (second external control) were detected only in pool 31, with a contamination rate of 0.09% (32 reads). The contamination threshold was, therefore, fixed at 0.09% of reads. No read of both the internal and external controls was detected in the water sample (negative control). No read from internal alien control and other detected viruses was detected in the external alien controls.

This survey detected a total of 23 virus species, among which two putative new virus species (belonging to *Totiviridae* and *Tymoviridae* families) and 21 already known species. Detected viruses belonged to 13 families and 15 genera (Table S7). Three virus families were detected in banana plants (*Betaflexiviridae*; *Caulimoviridae* and *Nanoviridae*) while the 10 others (*Caulimoviridae*; *Closteroviridae*; *Geminiviridae*; *Luteoviridae*; *Partitiviridae*; *Potyviridae*; *Solemoviridae*; *Tombusviridae*; *Tymoviridae*; *Totiviridae* and *Virgaviridae*) were detected in plants growing adjacent to banana plants.

Three banana viruses, BBTV, BanMMV, BSOLV, were confidently detected in banana samples (>0.09% contamination threshold and >10 reads) but also sometimes at very low number (>0.09% contamination threshold but <10 reads). BSOLV was reliably detected in one sample (Pool 22) only. Fewer than 10 reads (yet >0.09% contamination threshold) were detected for BSOLV, BSGFV, BSIMV, and BSMYV, corresponding to a non-confident detection. BSCAV and BSV-Lac reads were not detected. Importantly, no banana virus was detected in weeds and other crops samples; conversely, the viruses detected on other plants were not detected in bananas.

### 3.3. Comparison of Banana Virus Detection Between HTS Test on Pooled Samples and PCR-Based Tests on Individual Samples

IC-(RT)-PCR-based tests were previously carried out on individual banana samples (Table S2) [3]. The tests detected eight viruses: BBTV, BanMMV, and six BSV species (i.e., BSOLV, BSCaV, BSMYV, BSIMV, BSGFV, and BSV-Lac).

The VANA-HTS detected only three viruses: BBTV, BanMMV, and BSOLV. The VANA-HTS detected BanMMV with high confidence (35 to 2675 reads per sample) in all the 24 banana sample pools while the targeted RT-PCR test detected it in 41 individual sample(s) from 16 pools only (Figure S1; Table S2). Eight pools tested positive by HTS but negative by IC-RT-PCR came from Karonga, Chitipa, Dedza, and Mangochi districts. To analyze the discrepancies between IC-RT-PCR and HTS results, the sequences of the aligned reads on BanMMV genome for each sample were compared to the sequence of CP2 and CP9 primers used during targeted testing. This comparison highlighted mismatches between the generated sequences and the primer sequence (Tables S2 and S8A,B). There were generally two to three mismatches on both primers.

BBTV reads were detected in nine pools by the HTS, with a maximum of 183 reads. HTS detected BBTV with high confidence (reads number ≥ 10) in six pools (n°5, 9, 11, 12, 18, and 21) while the RT-PCR detected the virus in 29 individual samples from 16 pools (Figure S1, Tables S2 and S6). BBTV was detected by both techniques in seven pools (n°5, 8, 9, 11, 12, 18, and 19), among which pools 8 and 19 had low (<10 reads) confidence. BBTV was detected (10 reads) in Pool 21 (sample J3–11), for which individual samples were tested negative by IC-PCR. All the HTS BBTV detections with low (<10 reads (pools 7, 8, and 19)) were unreliable due to the too low number of reads. BBTV was detected by IC-PCR in individual samples from pools 1, 4, 6, 10, 13, 15, 16, 17, and 24 but not detected on pooled samples. Banana samples from Chitipa, Lilongwe, Mangochi, and Nsanje tested negative to BBTV in both diagnostic tests.

Among BSV species, the HTS test detected BSOLV with confidence (56 reads) in only one sample (Pool 22). Even if IC-PCR carried out on individual samples detected six BSV species (BSMYV, BSOLV, BSIMV, BSGFV, BSCAV and BSV-LAc) species in several samples, the very low number of reads (<10) originating from BSV impeded any relevant comparison between techniques.

### 3.4. New Host Detection

Chickpea chlorotic dwarf virus (CpCDV) and potato virus Y (PVY) were detected for the first time in a new host (and in Malawi for the first time): *Vigna unguiculata* (cowpea) for CpCDV and *Ipomoea batatas* (sweet potato) for PVY.

### 3.5. Identification and Characterization of Two New Viruses

#### Genome Sequence Reconstruction and Annotation

**Figure 3:**
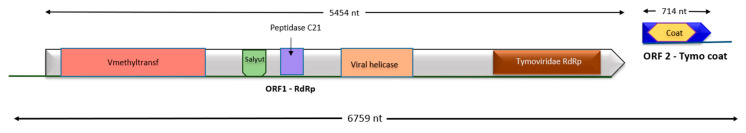
Genome organization of the novel virus of the family *Tymoviridae*. The long grey ORF represents ORF1 (RdRp in frame 1). Positions of the conserved sequence domains in the encoded proteins are indicated by colors: vmethyltransf (Pink color), Salyut (green color), Peptidase C21 (purple color), viral helicase (pale orange color), and the *Tymoviridae* RdRp (brown color). The ORF2 in dark blue color (coat protein in Frame 2) contains the Tymo-coat (yellow color) conserved domain.

Two novel viruses were identified in the sequencing datasets. In pool J3–15, contigs presenting homologies with *Tymoviridae* species were identified. BLAST (v. 2.16.0+) analysis showed that the closest hit was the Peach virus T (KY348614) (22% query cover and 69.1% nt identity). After contig extension, the nearly complete genome (6759 nt length) of a putative novel virus of the family *Tymoviridae* could be obtained (NCBI Acc. # PQ682664). This novel *Tymoviridae* genome contained two open reading frames (ORFs) comprising the RdRp and Tymo-coat conserved domains. The third ORF, the overlapping the polyprotein called movement protein, has not been identified and its start codon has not been sequenced as ORF (Figure 3). The first ORF is 5454 bp (1818 aa) long (positions 430 to 5883). The genome has the hallmarks of *Tymoviridae* including Vmethyltransf, Salyut, peptidase C21, Viral helicase, RdRp, and Tymo-coat. The Vmethyltransf. superfamily’s conserved domain (pfam01660, E-value = 4.42 × 10^−64^) was found in ORF 1 located between genomic position 544 and 1389. The other features detected in ORF 1 were the Viral_helicase1 domain (pfam01443, E-value = 1.85 × 10^−44^); the Peptidase C21 super family domain (cl05113, E-value = 1.31 × 10^−13^); and Salyut domain super family (cl41199, E-value = 3.18 × 10^−04^). The *Tymoviridae* RdRp domain (cd23247, E value 0 × 10^+00^) was detected between positions 4561 to 5682. The second ORF spans on 714 nt (from position 6023 to 6736; 238 aa). Tymo-coat super family (cl03052, E-value = 5.06 × 10^−25^) was found on this ORF between positions 6194 and 6661 (468 nt, 156 aa long).

**Figure 4:**
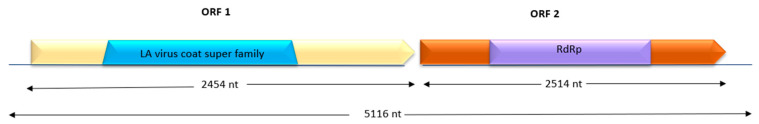
Genome organization of novel virus of the family *Totiviridae* with ORF1 (Coat protein) in orange and ORF2 (RdRp) in brown. Localisation of the conserved sequence domains in ORF1 and ORF2 are indicated by blue and pink colors, respectively.

In addition, a contig presenting homologies with *Totiviridae* species was identified in pool J3–16. BLAST analyses showed the contig was most closely related to *Pterostylis totivirus* (OL471348) with 70% nt identity (28% query cover). After contig extension, the near complete genome was obtained (5116 nt length, NCBI Acc. # PQ682663). This virus has two ORFs: LA-virus coat and RdRp (Figure 4).

The first ORF is 2454 nt (818 aa) long, and it is located between positions 55 and 2508 on the genome. This ORF encodes the coat protein with the LA-virus coat super family (cl07739, E-value = 2.50 × 10^−82^) motif located between 187–1380 nt (1194 nt; 398 aa long). The ORF second ORF was found from positions 2577 nt to 5090 nt (2514 nt and 838 aa long). This second ORF has the RdRp_4 domain (pfam02123, E-value = 1.48 × 10^−70^), located between position 2997–4424 (1428 nt and 476 aa long).

### 3.6. Phylogenetic Analyses

**Figure 5:**
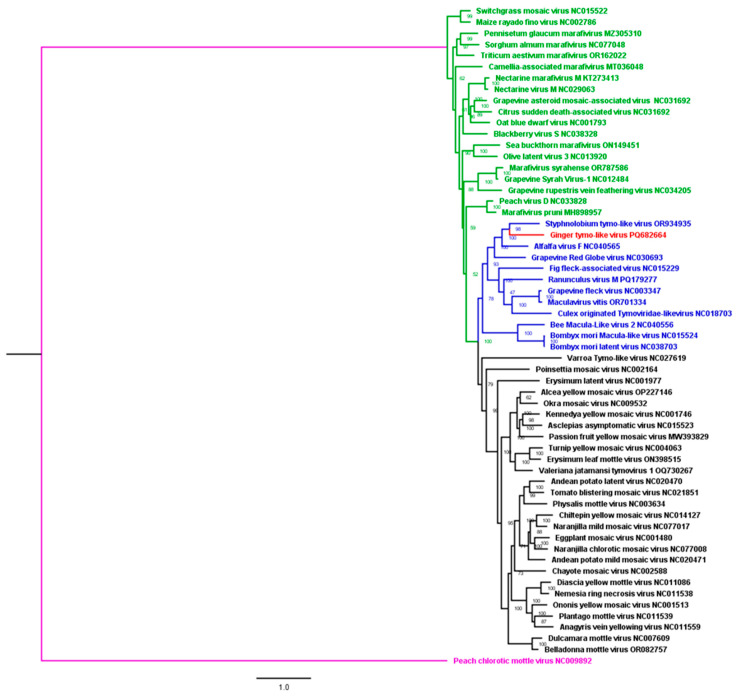
Maximum likelihood phylogenetic tree of nearly complete genomes of the new virus identified in this study with other viruses from the *Tymoviridae* family (green = *marafiviruses*; black = *tymoviruses*; blue = *maculaviruses*; and purple = root).

The genome pairwise and phylogenetic analyses of *Tymoviridae* genomes clustered the novel *Tymoviridae* in *Maculavirus* genus clade. The highest identity percentage were 50% and 48% with styphnolobium tymo-like virus (OR934935) virus and alfalfa virus F (NC_040565), respectively (Figure 5).

Phylogenetic trees for RdRp and CP amino acids sequences consistently clustered the novel *Tymorividae* in *Maculavirus* clade (Figure S2-1a,b). A pairwise matrix showed that the RdRp and the CP aa sequences of the novel species had a maximum of 57% and 35% identity with related species of the *Tymoviridae* family, respectively.

**Figure 6:**
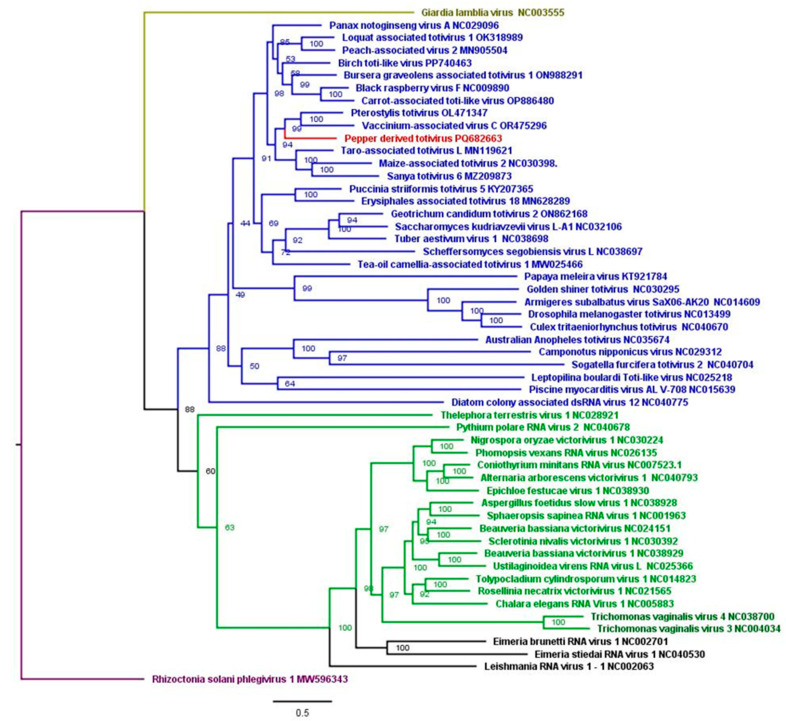
Maximum likelihood phylogenetic tree of nearly complete genomes of the new *Totivirus* identified in this study with other viruses from the *Totiviridae* family (blue = totiviruses; green = *victoriviruses*, black = *Leishmaniavirus*; brown = *Giardiavirus*; and purple = root).

Concerning the novel *Totiviridae* member, the phylogenetic analyses showed *pterostylis totivirus* isolate (OL471348) was the closest species with 52% nt identity (Figure 6). Pairwise identity analyses indicated that the novel totivirid CP shared a maximum of 52% aa identity with *pterostylis totivirus* isolates (OL471348), and its RdRp shared 65% aa identity with that of birch toti-like virus (PP740463). The phylogenetic trees showing novel totivirid CP did not cluster with any totivirus isolates that were used in tree construction (Figure S2-2a). For the RdRp, the closest sequence corresponded to the RdRp of the carrot associated toti-like virus (OP886480) (Figure S2-2b).

### 3.7. Confirmation by RT-PCR on Individual Samples

#### 3.7.1. Novel Viruses

**Figure 7:**
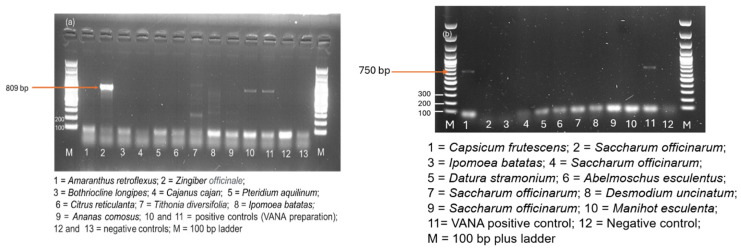
RT-PCR confirmation of (**a**) novel *Tymoviridae* and (**b**) novel *Totiviridae* detection on individual samples from the HTS pools.

The RT-PCR tests to detect the novel tymovirid species were performed on all the samples of the Pool 26 from Nkhata-Bay district (Table S3) using newly designed PCR primers. The novel virus was detected in only one sample corresponding to ginger (*Zingiber officinale*), and the results showed a positive band at expected size of 809 bp amplifying the Vmethyltransferase (Figure 7a). The PCR amplicon was sequenced (PV831853-54), and its identity was confirmed. Despite optimization of hybridization temperatures, some spurious bands of unexpected size were observed from *Tithonia diversifolia* and *Ipomoea batatas* (lines 7 and 8 on the gel).

In addition, another RT-PCR test was designed to detect the new *Totivirus* species. The novel virus, tentatively called pepper-derived totivirus, was confirmed in one hot pepper (*Capsicum frutescens*) sample collected in Zone 3 (Lilongwe district) (Figure 7b) and also sanger sequenced (PV930374-76).

#### 3.7.2. New Hosts for Viruses

Potato virus Y (PVY) was detected in sweet potato (*Ipomoea batatas*) for the first time by HTS, confirmed through targeted RT-PCR (Figure S3A) and Sanger sequencing (PV463391). Similarly, chickpea chlorotic dwarf virus (CpCDV) was identified in cowpea (*Vigna unguiculata*) using the same molecular technique and confirmed through targeted RT-PCR (Figure S3B) and its Illumina sequence accession number (PV829524). These results represent the first documented detections of PVY in sweet potato and CpCDV in cowpea, indicating new insights into the presence of these viruses in these crops.

#### 3.7.3. First Report of Nine Known Viruses in Malawi

In addition to PVY and CpCDV, the detection of five other viruses was confirmed by targeted RT-PCR and Sanger sequencing for the first time in Malawi. These species are pea seed-borne virus (PSbMV), pepper vein yellows virus (PeVYV), sweet potato leaf curl virus (SPLCV), citrus tristeza virus (CTV), and tomato mosaic virus (ToMV) (Figure S3C–G). The PCR products were sequenced and the sequences were deposited in the NCBI GenBank for SPLCV (PV463387-388), PVYV (PV463389-90), PVY (PV463391), ToMV (PV492510), and CTV (PV463392 and PV492511). The PSbMV Illumina sequences, PV829524 and PV829525, were deposited in the NCBI. The other two more viruses, Arhar cryptic virus (PV829522) and sugarcane bacilliform virus (PV829523), were detected for the first time, their sequences deposited in the NCBI but not confirmed by RT-PCR.

## 4. Discussion

### 4.1. Plant Sample Collection and Identification

In Malawi, like other African countries, bananas are mainly produced by smallholder farmers [37,38,39] who commonly intercrop bananas with other crops with the aim of effectively using available resources, and labor, increasing production per unit area of land, controlling soil erosion and minimizing climate change associated risks [40]. This study observed different field crops intercropped with bananas in Malawi. *Colocasia esculenta* (Taro), *Manihot esculanta* (cassava), *Saccharum officinarum* (sugarcane), and *Ipomoea batatas* (sweet potato) were commonly observed, in line with previous studies [41,42,43]. Nevertheless, few weed samples were collected in this study due to the scarcity of samples during the survey period. Indeed, the field survey was conducted in the summer (September 2020), which coincided with the period when farmers had cleared most fields to prepare for the next year’s crop sowing.

### 4.2. Sensitivity of HTS Tests on Pooled Samples

The inclusion of internal alien control theoretically allows for monitoring the test analytical sensitivity for each sample pool [29]. Nevertheless, to monitor properly the sensitivity, the number of reads on the internal alien target must be low, close to the limit of detection. In our case, the proportion of reads mapped to the three alien virus species was relatively high and variable (from 6% to 36% of generated reads in each sample). This high abundance is representing a limitation for the sensitivity of detection for low abundance viruses infecting the analyzed samples. This observation underlined the importance of evaluating the appropriate concentration of the alien targets to be added in the sample to monitor the sensitivity while not limiting it. Nevertheless, it is particularly challenging for virus enrichment protocol as the relative abundance of alien reads will also depend on the viral concentration in the analyzed samples, concentration that is unknown before sequencing. The first external alien control (Odontoglossum ring spot virus) was detected in five samples while external control 2 (Physostegia chlorotic mottle virus) contaminated two samples at rate of 0.01 and 0.09%, respectively. These percentages were very low and meant a theoretical contamination threshold (rate of 0.09%) below three reads for BanMMV (most abundant banana virus with a maximum of 2806 reads in a sample) and a single read BBTV (lower abundance with a maximum of 183 reads), respectively. The same threshold can be fixed for BSV species (one read) but, obviously, a detection cannot rely on a single read or two. Consequently, we used a threshold of 10 reads for considering a detection above the contamination background, whatever the virus.

### 4.3. The Results Comparison Between the HTS Test on Pooled Samples and PCR-Based Tests on Individual Samples

For banana samples, the VANA protocol applied on pools of samples confirmed the presence of some species already detected by targeted PCR (BBTV, BanMMV, and BSOLV) [3]. The results also confirmed the absence of CMV and BBrMV in Malawi. BanMMV was detected in pools where individual samples tested negative, confirming the highest inclusivity of HTS and the need to improve the inclusivity of current RT-PCR primers. Our findings concurred with the results obtained by Hanafi et al. [44] and Rong et al. [45] who observed false negatives in BanMMV infected samples tested by targeted RT-PCR tests used in this study. The observed mismatches between the generated reads and the primer sequences might have resulted to the non-binding of the primers to some sequences. The new detection of BanMMV by HTS protocol in Chitipa, and Karonga districts confirmed the widespread repartition of the virus, mostly asymptomatic when infecting a plant, in Malawi. Low titer of virus particles below the technique detection threshold in plant samples could be another reason that contributed to false detection of BanMMV by the IC-RT-PCR [46].

Samples positive for BBTV by IC-RT-PCR [3] were included in sixteen pools among which only five were considered as positive by the VANA protocol (>10 reads). For the samples detected only by IC-RT-PCR, the putative low concentration of BBTV combined with a low prevalence in some pools [3] and the high abundance of internal control alien virus could, therefore, explain the false negative in these pooled samples. On the other hand, BBTV was detected by HTS test in Pool 21 (10 reads), while no PCR test was positive. The origin of the BBTV detection in this sample might be due to technical and biological limitations but is unclear as the primer sequences were not covered by the reads. Anyway, the number of BBTV reads was always low in the analyzed samples, limiting potentially the analytical sensitivity of the test. Another HTS protocol, based on the high throughput sequencing of ribodepleted total RNA, led to the same observation of a low number of reads and high false negative risk for BBTV [45]. A protocol based on the rolling circle amplification of circular DNA might deliver better detection of BBTV, as seen for many geminiviruses [47].

A similar situation has been observed for BSV species with always a very small quantity of reads in the datasets, except in one sample for BSOLV. The low number of reads suggested that our VANA protocol applied on pooled banana samples has very low sensitivity to BSV species. Overall, our VANA results concurred with the findings of Schönegger et al. [14] who used VANA and detected BBTV and BSOLV by very low read numbers. Low sequencing depth in HTS could have stemmed from the low initial viral load in the plant tissues putatively coupled with the low efficiency of sample preparation from banana leaves. Indeed, VANA was proposed as a convenient HTS-based protocol for detecting viruses from plants having high levels of either phenolic compounds or viscous polysaccharides [48], but has not been systematically tested on banana leaves, another plant known to be rich in phenolic compounds which could interfere with the proper sample preparation [49]. These results, together with the high false positive rate of BSV detection and low sensitivity for BBTV of another HTS protocol based on ribodepleted total RNA sequencing [45], advocate for the IC-PCR as preferred protocol for detecting BBTV and BSV in banana samples.

### 4.4. The Absence of Virus Exchange Between Banana and Surrounding Plants Deserve Complementary Surveys

Our results indicated that the sampled plants surrounding banana plants were not infected by banana viruses. In this study, ten plants of taro (*Colocasia esculenta* L.) in the vicinity of BBTV infected banana plants were analyzed but determined as negative to BBTV and other banana viruses. Our findings contrasted with the findings of Pinili et al. [50] and Vikrant et al. [51], who detected BBTV in taro. Our results can originate from differences in taro cultivar susceptibility to BBTV, as suspected by Pinili et al. [50], or to a very low incidence of BBTV on taro, hardly sampled and detectable with only 10 samples. Although our results suggested that the virus exchange can be low or absent, a robust conclusion, important to manage BBTV epidemics, would need the testing of many more samples around specific spots. The low number of reads obtained for BBTV suggested also a limited sensitivity and the complementary survey should preferably be carried out using targeted IC-PCR tests.

Some viruses can be latent in the wild or cultivated plant species [49] while posing a threat to another crop species [52]. For example, recent studies detected cactus virus X (CVX) and sugarcane bacilliform Guadeloupe A virus-2 (SCBGAV-2) in banana plants [24,53]. Our study did not detect any virus infecting surrounding plants in asymptomatic banana plants, suggesting the absence or the very low occurrence of transfer of viruses from these species to banana (due to host compatibility or lack of transmission pathway).

### 4.5. Novel Hosts for Known Viruses

Our study adds a new host, sweet potato from *Convolvulaceae*, in the large host-range of potato virus Y, that already includes crops and wild hosts from twelve plant families (*Amaranthaceae*, *Asteraceae*, *Brassicaceae*, *Boraginaceae*, *Chenopodiaciae*, *Cucurbitaceae*, *Euphorbiaceae*, *Geraniaceae*, *Lamiaceae*, *Malvaceae*, *Moringaceae*, and *Solanaceae*) [54]. This detection, independently confirmed by RT-PCR and also representing its first detection in Malawi, adds also a new family in the host range of potato virus Y [54].

Chickpea chlorotic dwarf virus (CpCDV) belongs to the *Geminiviridae* family and has a large host range including species from *Fabaceae*, *Asteraceae*, *Amaranthaceae*, *Brassicaceae*, *Cucurbitaceae*, *Caricaceae*, *Chenopodiaceae*, *Leguminosae*, *Malvaceae*, *Pedaliaceae*, and *Solanaceae* families. However, our results are the first detection in cowpea (*Vigna unguiculata* [L.] Walp), representing an additional host within the *Fabaceae* family [55].

### 4.6. A Novel Tymoviridae Species

Our putative new tymovirid has a nearly complete genome of 6.8 kb within the length range (6.7 kb to 7.5 kb) of *Maculaviruses*.

For genome and CP aa identities, the ICTV species demarcation criteria for the *Tymoviridae* family corresponds to less than 80% for the genome and less than 90% for the CP aa sequences. The highest genomes nt identity to our novel virus was 50% (*Styphonolobium* tymo-like virus—OR934935) and the highest aa identity for CP was 28% (grapevine red globe virus—NC030693). These results were far below the demarcation threshold and therefore suggested that the detected genome belongs to a new species, tentatively named ginger tymo-like virus (GTLV). The genome of this putative new species has two separated ORFs, like culex tymoviridae-like virus (CuTLV) [56], while the marafivirus genomes possess two extensively overlapping ORFs and *Maculaviruses* possess four ORFs. *Tymovirus* and *Marafivirus* genuses members possess a conserved sequence that serves as a sgRNA promoter known as the “tymobox” and “marafibox” but not the *Maculavirus* member (ICTV) [57]. The novel virus is devoid of sgRNA promotor. The first ORF (the longest longest) translates the RNA dependent RNA polymerase (RdRP) and the second ORF, the Tymo-coat. It was noted that the novel GTLV shares similarities with other species by possessing methyltransferase domain, viral helicase, salyut domain super family, peptidase, RdRp domain, and coat protein.

In addition, it is the first report of *tymovirid* in *Zingiberaceae* species. *Tymoviridae* members have very narrow host ranges, reinforcing the hypothesis of a new species, and *Maculavirus* are strictly confined to the phloem of infected hosts, causing disease in grapevine, alfalfa, and figs [58,59,60,61]. Some members of this genus infect insects such as bee (*Apis mellifera*) with bombyx mori virus [58], *Helicoverpa armigera* and *Trichoplusia* with bombyx mori latent virus [58,59], and mosquito (*Culex quinquefasciatus*) (culex originated *Tymoviridae*-like virus) [59]. *Maculaviruses* are not transmitted through seeds [60] but through infected propagative materials. No vector has been identified so far in any species, but field infection can spread [62] and transmission of *Maculaviruses* through dodder was also discovered by Woodham and Krake [63].

### 4.7. A Novel Totiviridae Virus Species

For totiviruses, biological criteria, especially host species, are important demarcation criteria. Some totiviruses infect fungi while others infect plants. Black raspberry virus, carrot associated toti-like virus, loquate associated totivirus, maize-associated totivirus, peach-associated virus 2 and taro associated totivirus are examples of *Totiviruses* reported to infect economically commercial crops. This is the first report of a totivirid derived from pepper, its genome sequence clustered with plant viruses although its exact host (pepper or a fungus infecting pepper) should be determined.

The length of the nearly complete genome of our novel totivirid is 5181 nt, which is within the range of the totivirus (4500 nt to 7000 nt) [63]. The genome of *Totiviruses* usually contains two large, usually overlapping, ORFs: the 5′-proximal ORF encodes the major CP (Gag) and the 3′-proximal ORF encodes an RdRp (ICTV, 2024, https://ictv.global/report_9th/dsRNA/Totiviridae, accessed on 23 October 2022). The novel totivirid has two large ORFs, like tuber aestivum virus 1 (NC_038698), the first ORF encodes CP and the other one the RdRp domain.

The host is a prime criterion used by ICTV to differentiate *Totiviridae* members (ICTV, https://ictv.global/report_9th/dsRNA/Totiviridae, accessed on 23 October 2022). Since novel totivirid was detected for the first time in pepper it might be qualified as a novel virus which could be named pepper-derived totivirus (PDTV). Additionally, though it is not commonly used, sequence identity at the protein level generally reflects a species difference, especially if they share less than 50% sequence identity with each other. The pairwise analyses of the genome, the CP aa and RdRp aa of our new virus, revealed a maximum identity with known totivirus species of 39% (Loquat-associated totivirus OK318989) and 58% (Taro-associated totivirus, MN119621), respectively. Totiviruses are transmitted during cell division (ICTV, 2024).

Further studies on the two novel viruses would investigate their symptomatology or association with symptoms, if any, during field surveys, their host range, and symptom severity in greenhouse experiments, provided a transmission means is identified. Overall, the phytosanitary interest of these complementary studies should be envisioned according to a recently proposed framework for the prioritization of biological characterization of newly discovered viruses according to the risk they can potentially cause [36].

## 5. Conclusions

This study added two new viruses, ginger tymo-like virus and pepper-derived totivirus to the scientific world. It has also unveiled sweet potato and cowpea as new hosts for potato virus Y and chickpea chlorotic dwarf virus (CpCDV), respectively. These findings not only expand known host ranges but also signal potential emerging threats to local food security and trade. Confirmation of these new host associations underscores the importance of continued surveillance and accurate diagnostic tools to monitor viral spread and guide mitigation strategies. The presence of known and potentially novel viruses, alongside new host–virus associations, calls for strengthened SPS protocols, particularly in germplasm exchange, regional trade, and seed system development, to prevent the introduction and dissemination of harmful viruses within and beyond banana production systems. This study underlined some advantages and limitations of the use of HTS technologies on pooled samples for large scale virome survey. The better inclusivity observed for some viruses like BanMMV is counterbalanced by a low sensitivity for BBTV and the BSV species. The observed limitations should warrant a careful evaluation of protocol selection for further surveys, whatever the plant species. The high inclusivity of the protocol is also showcased by the new hosts and new country detection for nine known virus species and the discovery of two new plant hosts. For banana viruses, the HTS protocol did not detect any new virus that could cause high epidemics risks. Concerning the BBTV epidemics, further investigations should be carried out in Chitipa, Mangochi, and Nsanje, which are so far the only districts where the virus has not been detected to confirm their status of BBTV-free areas that might be useful in the establishment of nurseries to deliver BBTV-free plantlets locally and nationally.

## Data Availability

Research data was deposited in the SRA (BioProject: PRJNA1193526) and can be accessed on: https://dataview.ncbi.nlm.nih.gov/objects?linked_to_id=PRJNA1193526&archive=biosample (accessed on 23 July 2025).

## Supplementary Materials

The following supporting information can be downloaded at: https://www.mdpi.com/article/10.3390/v17081068/s1, Method S1. Nucleic acid (RNA) extraction, and RT-PCR protocol; Figure S1. Comparision of HTS and RT-PCR results; Figure S2-1(a-b). Tymoviridae CP and RdRp phylogenetic trees; Figure S2-2(a-b). Totiviridae CP and RdRp phylogenetic trees; Figure S3(A-G). RT-PCR virus confirmatory test results; Table S1. Plant samples, their family and species names; Table S2. Combined IC-(RT)-PCR; the HTS results and primers mismatch analysis; Table S3. High throughput sequencing pools, host plants and their symptomatic status; Table S4. Tymoviridae and Totiviridae reference genomes from NCBI; Table S5. viruses tested by RT-PCR on plants and their primers; Table S6. HTS with host names (banana and other plants) and pool numbers; Table S7. Virus name, its family and genus names, their associated host plants and the collected sites; Table S8A. Nucleotide mismatches analysis between CP2 primer and BanMMV consensus sequence; Table S8B. In depth mismatch analysis of banana mild mosaic virus sequences and BanMMV CP 9 primers.
