# Supplementary material for: Virome Survey of Banana Plantations and Surrounding Plants in Malawi
Source: Viruses. 2025 Jul 31;17(8):1068. doi: 10.3390/v17081068 (PMC12390665; doi:10.3390/v17081068)
Supplement: Supplementary file 1 [file viruses-17-01068-s001.zip › Figure S1. Comparision of HTS and RT-PCR results.pdf]

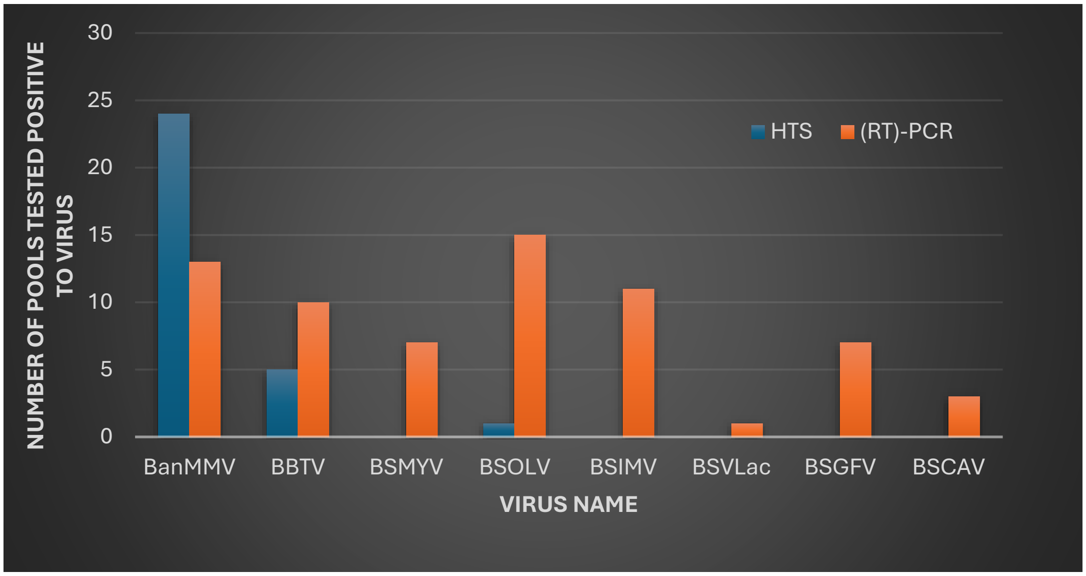

Figure S1. Comparison of HTS and RT-PCR sensitivity in virus detection on banana leaf samples. HTS in blue and RT-PCR in pink coulor, the bar charts present the number of banana pools in viruses (BanMMV, BBTV, BSMYV, BSOLV, BSIMV, BSVLAC, BSGFV and BSCAV were detected.
