# Supplementary material for: Virome Survey of Banana Plantations and Surrounding Plants in Malawi
Source: Viruses. 2025 Jul 31;17(8):1068. doi: 10.3390/v17081068 (PMC12390665; doi:10.3390/v17081068)
Supplement: Supplementary file 1 [file viruses-17-01068-s001.zip › Figure S2-1(a-b). Tymoviridae CP and RdRp phylogenetic trees.pdf]

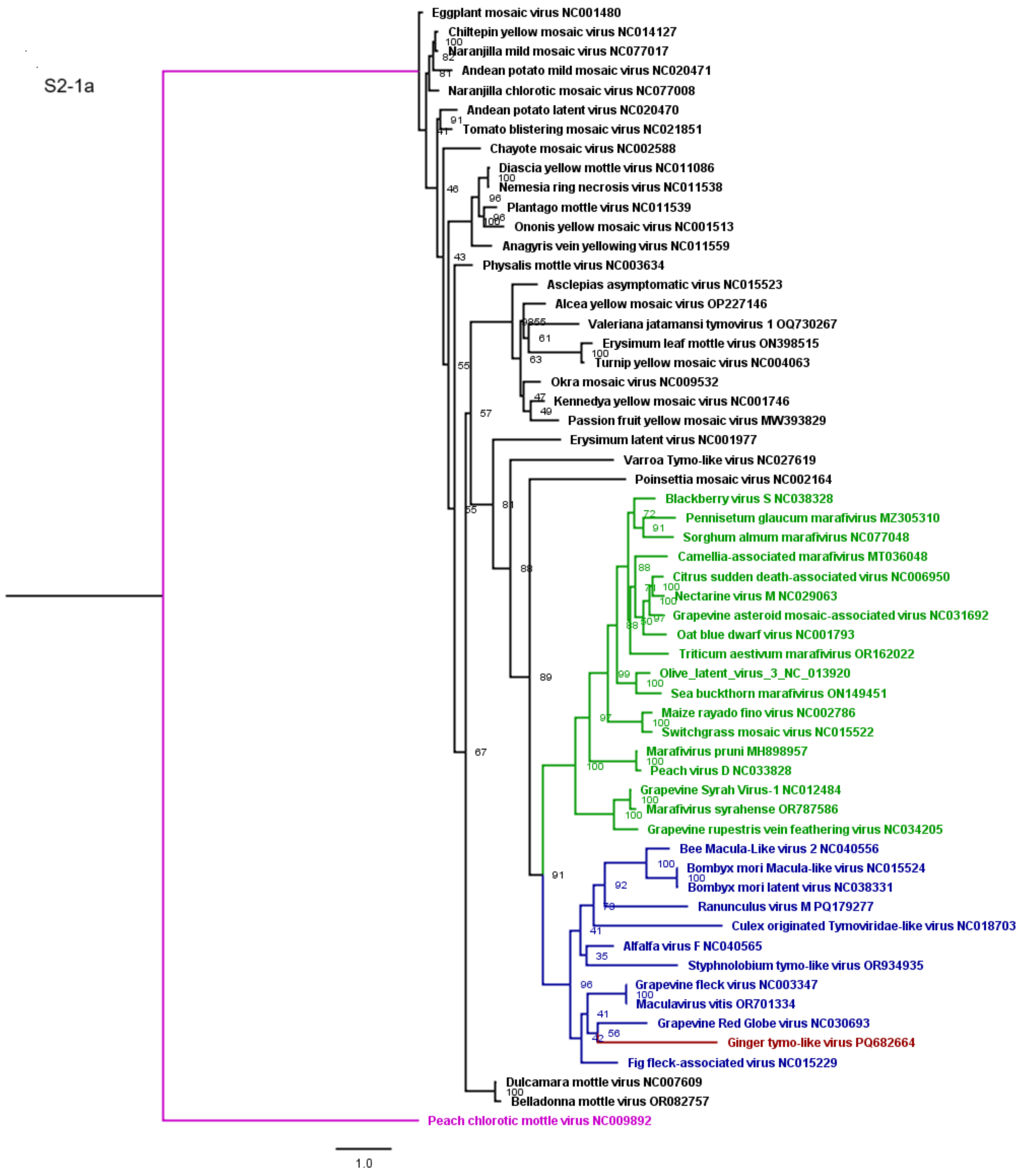

Figure S2-1a. Tymo-coat protein phylogenetic tree of the new viruses identified in this study with other viruses from the same families (green = marafiviruses; black = tymoviruses; blue = maculaviruses and purple = root)

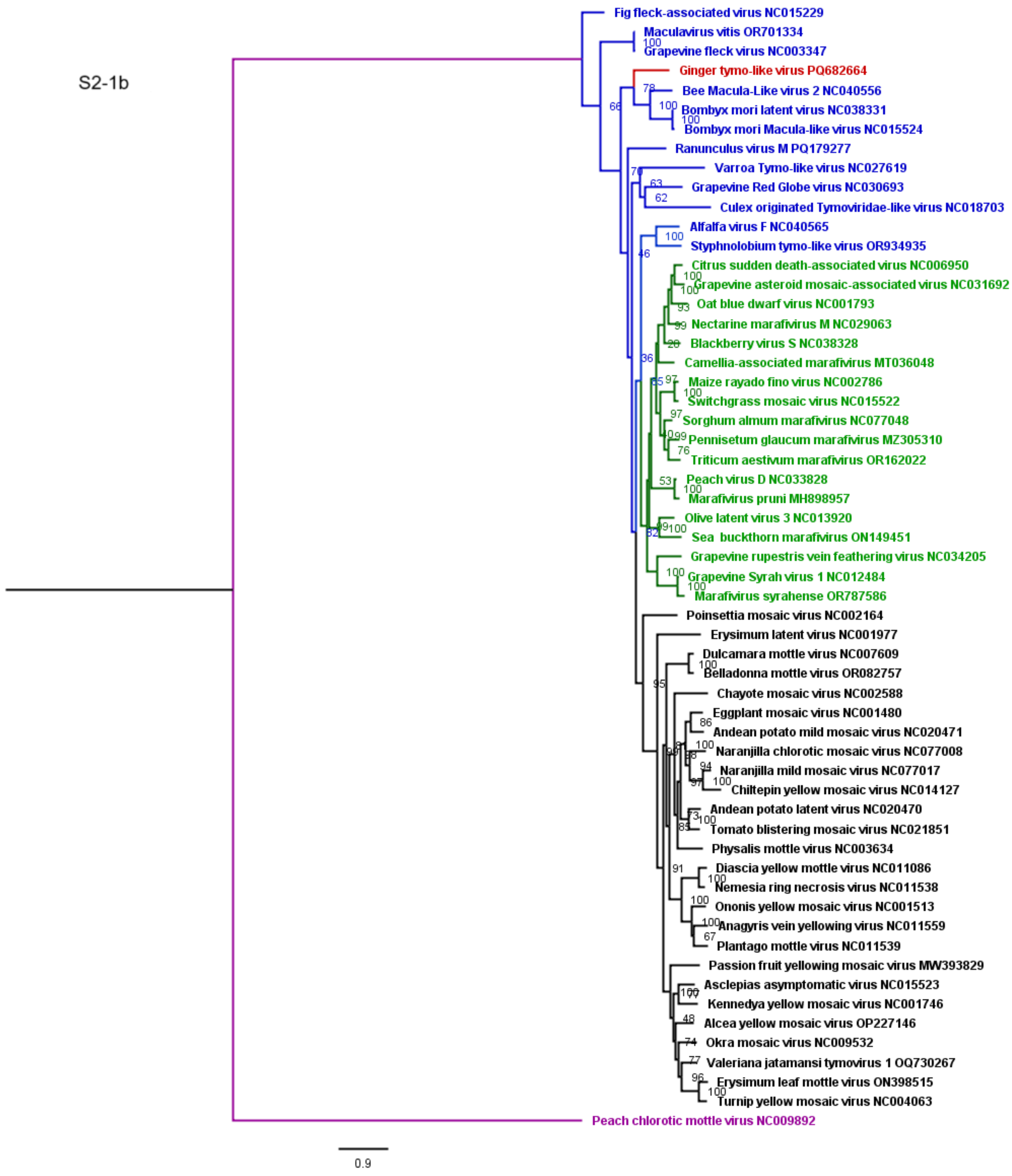

Figure S2-1b. Tymoviridae RdRp amino acid phylogenetic tree of the new viruses identified in this study with other viruses from the same families (green = marafiviruses; black = tymoviruses; blue = maculaviruses and purple = root)
