# Supplementary material for: Virome Survey of Banana Plantations and Surrounding Plants in Malawi
Source: Viruses. 2025 Jul 31;17(8):1068. doi: 10.3390/v17081068 (PMC12390665; doi:10.3390/v17081068)
Supplement: Supplementary file 1 [file viruses-17-01068-s001.zip › Figure S2-2(a-b). Totiviridae CP and RdRp phylogenetic trees.pdf]

S2-2a

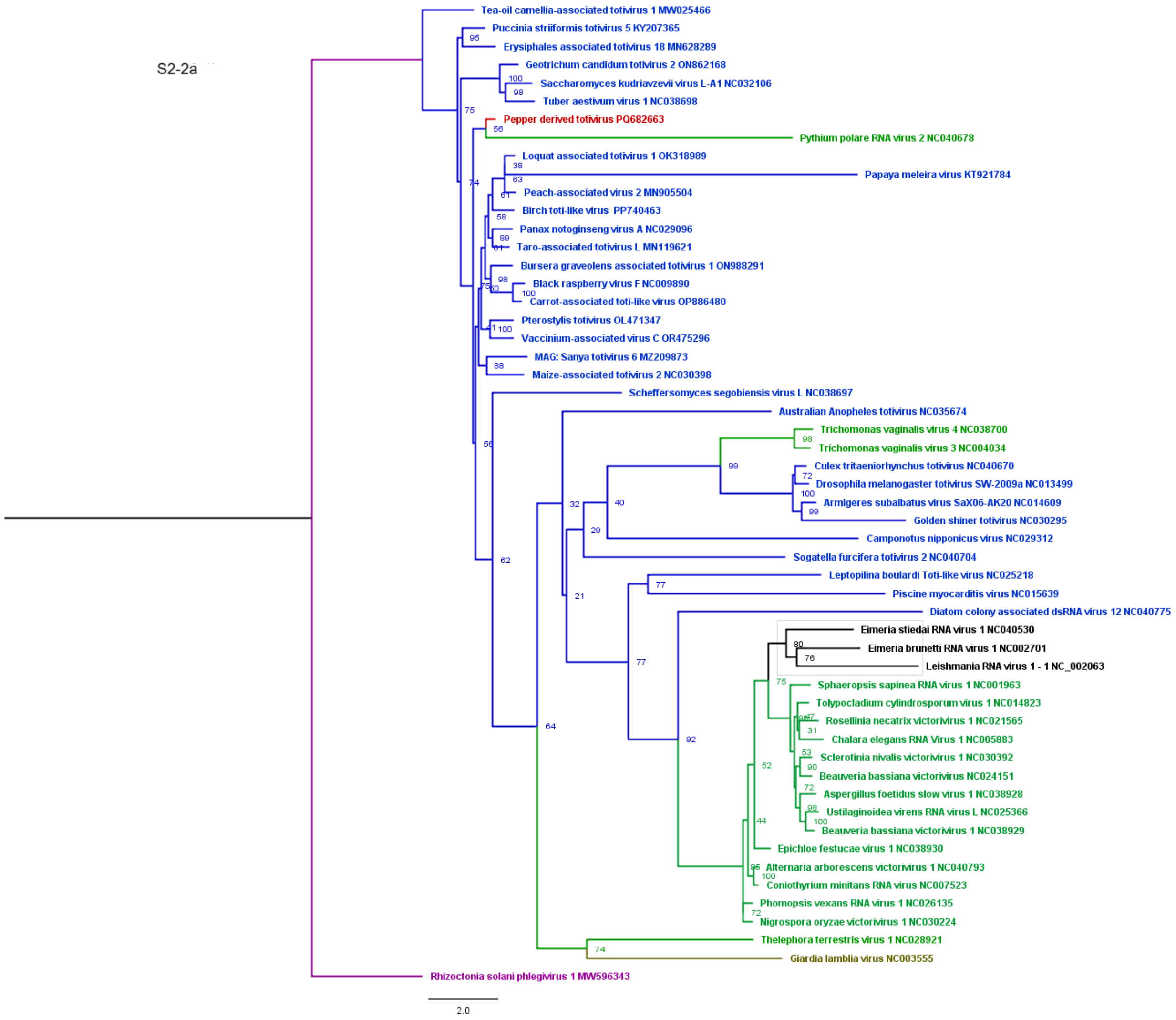

Figure S2-2a. Totiviridae coat proteins phylogenetic tree of the new viruses identified in this study with other viruses from the same families (green = Totiviruses; black = Victoriviruses, light blue = Leishmaniavirus; brown = Giardiavirus and purple = root)

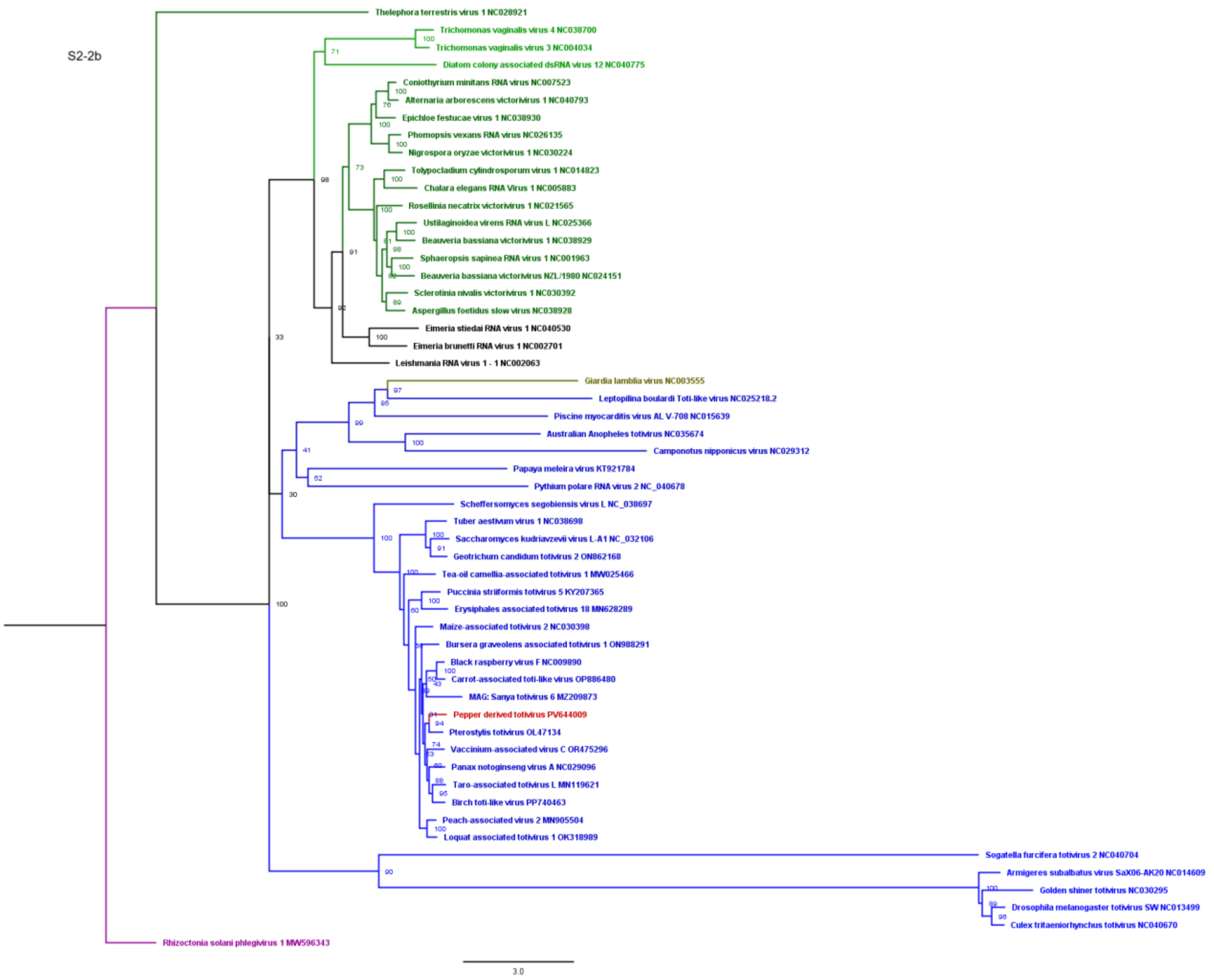

Figure S2-2b. Totiviridae RdRp amino acids of the new viruses identified in this study with other viruses from the same families phylogenetic tree (green = totiviruses; black = victoriviruses, light blue = Leishmaniavirus; brown = giardiavirus and purple = root).
