# Supplementary material for: Virome Survey of Banana Plantations and Surrounding Plants in Malawi
Source: Viruses. 2025 Jul 31;17(8):1068. doi: 10.3390/v17081068 (PMC12390665; doi:10.3390/v17081068)
Supplement: Supplementary file 1 [file viruses-17-01068-s001.zip › Figure S3(A-G). RT-PCR virus confirmatory test results.pdf]

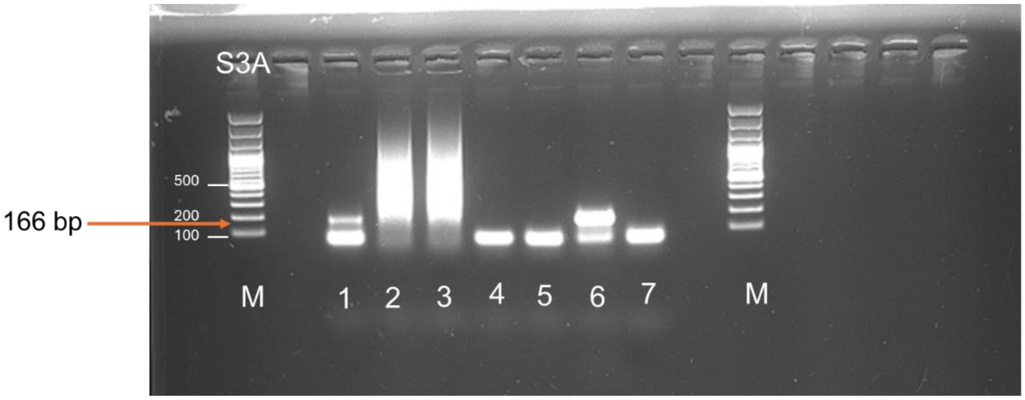

1 = *Ipomoea batatas* from Rumphi (Pool 26);
2-3 = product PCR for samples from Pool 26;
4-5 = *Ipomoea batatas* from Nkhotakota (Pool 27);
6 = VANA positive control; 7 = Negative control; M = 100 bp ladder

Figure S3A: Gel picture for the RT-PCR detection test targeting Potato virus Y

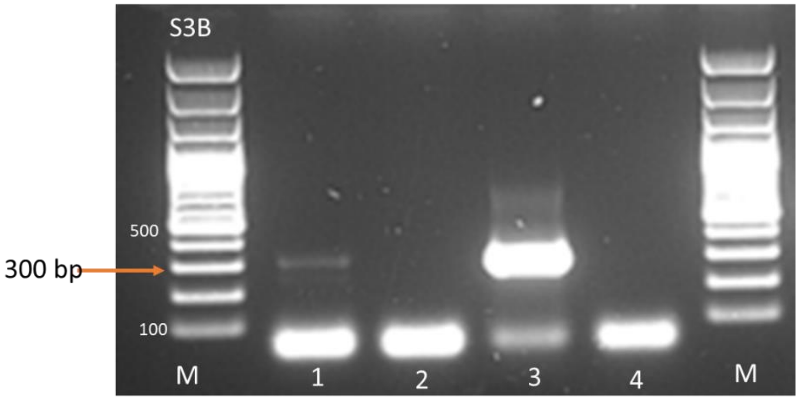

1 = *Vigna unguiculata*; 2 = *Colocasia esculenta*;
3 = VANA positive control; 4 = negative control
M = 100 bp plus ladder

Figure S3B: Gel picture for the RT-PCR detection test targeting the chickpea chlorotic dwarf virus

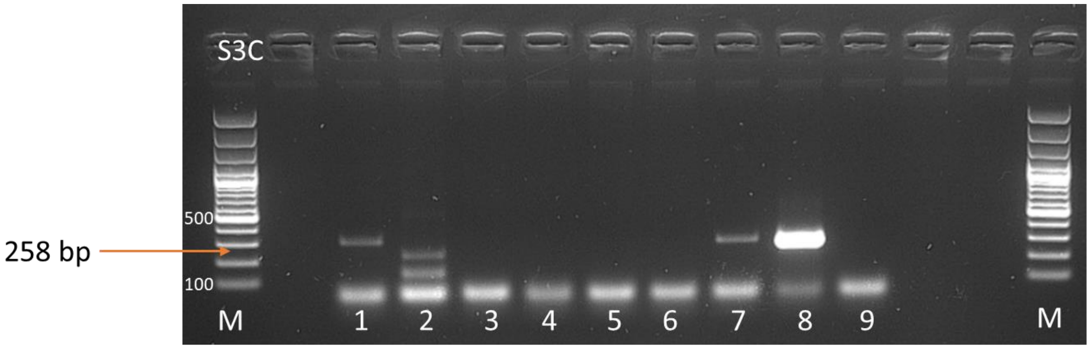

1 = *Citrus reticulanta*; 2 = *Capsicum frutescens*; 3 = *Saccharum officinarum;*
4 = *Ipomoea batatas*; 5 = *Saccharum officinarum;* 6 = *Citrus reticulanta*;
7 = *Citrus aurantium*; 8 = VANA positive control; 9 = negative control;
M = 100 bp plus ladder

Figure S3C: Gel picture for the RT-PCR detection test targeting the Citrus tristeza virus

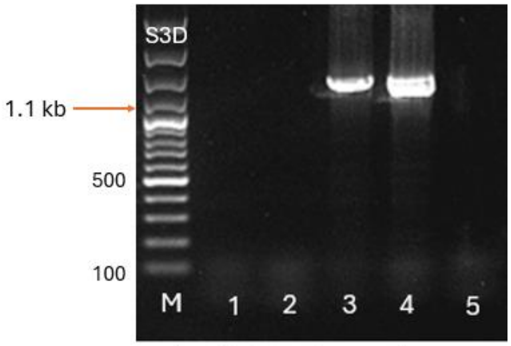

1 = *Datura stramonium*;
2 = *Solanum lycoperscium;*
3 = *Capsicum frutescens;*
4 = VANA positive control;
5 = negative control;
M = 100 bp plus ladder

Figure S3D. Gel picture for the RT-PCR detection test targeting the pepper vein yellows virus

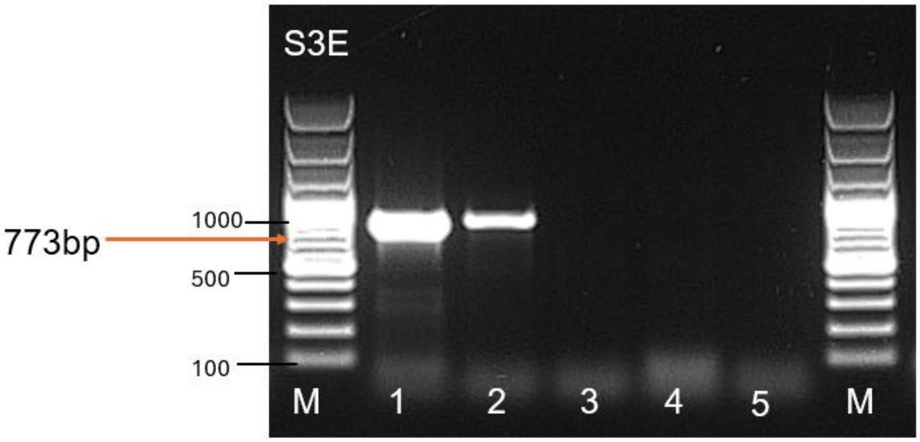

1 = *Ipomoea batatas* from Rumphi (pool 26);
2 = *Ipomoea batatas* from Nkhotakota (pool 27);
3 = *Ipomoea batatas* from Phalombe (pool 29);
4 = *Ipomoea batatas* from Chikwawa (Pool 31);
5 = negative control; M = 100 bp plus ladder

Figure S3E. Gel picture for the RT-PCR detection test targeting the sweet potato leaf curl virus

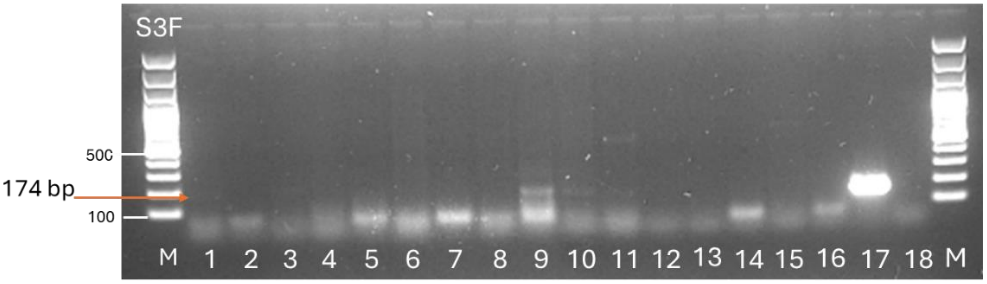

1 & 2 = *Annona squamosa*; 3 & 4 = *Zea mays*; 5 & 6 = *Cymbopogon citratus*;
7 & 8 = *Saccharum officinarum*; 9 &10 = *Solanum lycopersicum*;
11 & 12 = *Carica papaya*; 13 & 14 = *Ipomoea batatas*; 13 & 14 = *Vigna unguiculata;*
15 & 16 = *Manihot esculenta*; 17 = VANA positive control; 18 = negative control;
M = 100 bp plus ladder

Figure S3F. Gel picture for the RT-PCR detection test targeting the tomato mosaic virus

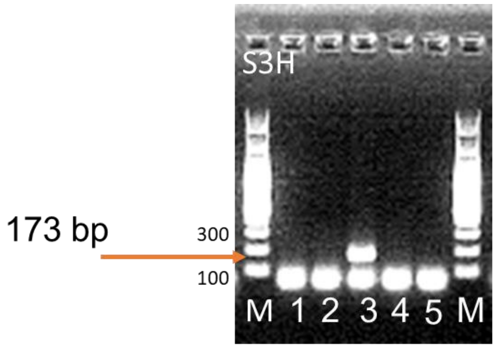

1 = *Phaseolus vulgaris*
2 = *Phaseolus vulgaris*
3 = *Pisum sativum*
4 = *Vicia faba*
5 = negative control
M = 100 bp ladder

Figure S3G. Gel picture for the RT-PCR detection test targeting Pea seedborne virus
