# Supplementary material for: Virome Survey of Banana Plantations and Surrounding Plants in Malawi
Source: Viruses. 2025 Jul 31;17(8):1068. doi: 10.3390/v17081068 (PMC12390665; doi:10.3390/v17081068)
Supplement: Supplementary file 1 [file viruses-17-01068-s001.zip › Method S1. Nucleic acid (RNA) extraction, and RT-PCR protocol.pdf]

Method S1

1.0 Nucleic acid (RNA) extraction protocol

The working benches and pipettes used in this task were first cleaned with 0.1 M NaOH solution, whipped with clean tissue and absolute ethanol as final step. The nucleic acid (RNA) extraction was done using the Qiagen kit (https://www.qiagen.com/us/products/discovery-and-translational-research/dna-rna-purification/rna-purification/total-rna/rneasy-kits) and followed manufacturers' instructions with minor modifications. Briefly, samples were flash-frozen in liquid nitrogen by dipping the extraction bag part containing leaf samples in liquid nitrogen until no bubbles were seen. The frozen leaf samples were quickly grounded into fine powder using a homogenizer. 1200 µl (1.2 ml) of cold buffer RLT Plus and was immediately added to the plant powder before it thawed. Six hundred microliters of crude extracts were transferred into a labelled gDNA Eliminator spin column placed in a 2 ml collection tube. Samples were kept in a foaming box with ice and up until all samples had undergone the above steps. Samples were then centrifuged for 30 s at ≥8000 g (≥10,000 rpm) at 4°C. The columns were discarded, and the flow throughs were saved in a new 1.5 ml collection tube (supplied by RNeasy® Plus Mini Kit). 1 volume of 70% ethanol was to the flow-through and mixed well by pipetting.

Six hundred microliters of each mixture (sample + alcohol), were transferred into RNeasy spin column placed in a 2 ml collection tube. This was followed by the centrifugation for 15 s at ≥8000 g (≥10,000 rpm) at 4°C. The flow-through were discarded. 700 µl Buffer RW1 was added to each RNeasy Mini spin columns (in a 2 ml collection tube, supplied

by RNeasy® Plus Mini Kit) and centrifuged for 15 s at ≥8000 g (≥10,000 rpm) at 4°C. The flowthroughs were discarded. 500 µl Buffer RPE was added to the RNeasy spin column and centrifuged for 15 s at ≥8000 g (≥10,000 rpm) at 4°C followed by the discarding of the flow-through. Another 500 µl Buffer RPE to the RNeasy spin column and centrifuged for 2 min at ≥8000 g (≥10,000 rpm) at 4°C. RNeasy spin columns were placed in a new 2 ml collection tubes (supplied by RNeasy® Plus Mini Kit) and centrifuged at full speed for 1 min at 4°C to further dry the membrane. The RNeasy spin columns were then placed in a new 1.5 ml collection tubes (supplied by RNeasy® Plus Mini Kit). 50 µl RNase-free water added directly to the spin column membrane, and centrifuged for 1 min at ≥8000 g (≥10,000 rpm) at 4°C to elute the RNA. All the 1.5 ml collection tubes were carefully marked with a marker with the name of each sample and extraction data.

The eluted RNA samples were immediately put on ice in the foaming box followed by quality assessment on the Nanodrop. The extracted RNA for each sample was aliquoted and kept at -80°C for future usage.

2.0 Reverse transcription polymarase chain reaction (PCR)

2.1 Reverse transcription (RT)

The RNA samples were put on ice. The thermal cycler was pre-heated to 65°C.

The Master Mix 1 for the first strand was prepared as in table 1 below.

Table 1 - Master Mix 1:

**Reagents**	**Volume/reaction**
PCR Water	9 µl

10 mM dNTPs	1 µl
50 µM Random hexamer	1 µl
RNA sample	2 µl
**Total volume for one reaction**	**13 µl**

The number of reactions was calculated based on the number of samples passing the RNA quality control plus a negative control. The mixture was heated for 5 min at 65°C and immediately incubated on ice for at least 1 min.

The second strand was synthesized by first preparing a second Master Mix 2 comprised of 4 µl of 5x First-Strand Buffer, 1 µl of 0.1M DTT, 1 µl of RNaseOUT^{TM} and 1 µl SuperScript III RT^{TM} (200 units/µl) (total volume 7 µl per sample) which was added to the first mix and incubated in the thermal cycler at 25°C for 5 min, then 50°C for 60 min and finally at 70°C for 15 min (https://assets.thermofisher.com/TFS-Assets/LSG/manuals/superscriptIIIfirststrand_pps.pdf (accessed July 10 2023). The reaction was stopped by putting the tubes directly on ice for at least 1 min. The synthesized cDNAs were then either kept at 4°C (max 4 weeks) or stored at -20°C for future usage.

2.2 Polymerase chain reaction (PCR)

The synthesized cDNAs were then amplified using 2.0 µl in a 25 µl reaction containing 5.0 µl 5x Mango Taq buffer; 2.0 µl 50nM $MgCl_2$, 0.5 µl dNPTs mix, 13.25 µl PCR water, 0.25 µl Taq polymerase and 1 µl of each primer (reverse and forward). The thermal cycling was: 94 °C for 5 min, then 40 cycles of 94 °C/20 s, Ta/30 s, 72 °C/60 s then a single stage 72 °C for10 min for final extension and then end with an indefinite hold at 4 °C. Ta is the hybridization temperature which varied according to the primers. The PCR

products were analyzed by electrophoresis on a 1% agarose gel in Tris-acetate-EDTA buffer, stained with GelRed nucleic acid gel stain (Biotium), and visualized under UV light.

3.0 Amplification of the plant DNA.

This was done on plant materials that tested positive to viruses but its quality degraded after long storage time. The nucleic acids extraction and cDNA preparation were executed as stipulated above. The amplification of the plant DNA was done using the 81s primers. The 18S ribosomal RNA primers (Forward = 5' TTC-CAT-GCT-AAT-GTA-TTC-AGA-G 3' and Reverse =5' ATG-GTG-GTG-ACG-GGT-GAC 3') were used to amplify the plant DNA. The master mix was prepared by following the protocol provided in Table 2.

Table 2 Master mix

**PCR Reagents**	**Brand**	**µl/reaction**
5x Mango Tag reaction buffer	Bioline	5.00
50 mM $MgCl_2$	Bioline	0.75
10 mM dNTP mix	Eurogentec	0.50
F Primer (25 µM)	Eurogentec	0.50
R Primer (25 µM)	Eurogentec	0.50
Mango Taq (5U/µl)	Bioline	0.5
DNA		1.0
dNase/Rnase free water		16.25
Total volume		25.00

The reactions were incubated in the thermocycler at the following condition, 94 °C for 5 min, then 35 cycles of 94 °C/20 s, aT(°C)/30 s, 72 °C/60 s then a single stage 72 °C for10 min for final extension and then end with an indefinite hold at 4 °C. The expected PCR product size was 459 bp was amplified.

Sanger sequencing

The amplicon were isolated from the gel, cut and purified using the Macherey-Nagel offers a comprehensive range of DNA purification kits (https://www.xn--mnnet-

hu3b.com/bioanalysis/kits/dna/) under their NucleoSpin® by following the manufacturer's instructions. Sequencing was done by the Macrogen Europe (Netherland).
