# Supplementary material for: Virome Survey of Banana Plantations and Surrounding Plants in Malawi
Source: Viruses. 2025 Jul 31;17(8):1068. doi: 10.3390/v17081068 (PMC12390665; doi:10.3390/v17081068)
Supplement: Supplementary file 1 [file viruses-17-01068-s001.zip › Table S1. Plant samples, their family and species names.pdf]

Table S1. Collected plant samples, their pools, locations and individual numbers; family and species names. This table has pools numbers, number of samples in a pool, local names of samples, sample family and species names and also classified weather a sampled plant was a crop or weed.

| **Pool #** | **Location** | **Sample number** | **Local or common plant name** | **Family** | **Species** | **Type** |
|---|---|---|---|---|---|---|
| 1 | Chitipa and Karonga | 12 | Banana | *Musaceae* | *Musa species* | Crop |
| 2 | Chitipa | 9 | Banana | *Musaceae* | *Musa species* | Crop |
| 3 | Chitipa | 9 | Banana | *Musaceae* | *Musa species* | Crop |
| 4 | Karonga | 13 | Banana | *Musaceae* | *Musa species* | Crop |
| 5 | Nkhatabay, Nkhotakota and Rumphi | 11 | Banana | *Musaceae* | *Musa species* | Crop |
| 6 | Nkhatabay | 11 | Banana | *Musaceae* | *Musa species* | Crop |
| 7 | Nkhotakota | 14 | Banana | *Musaceae* | *Musa species* | Crop |
| 8 | Nkhatabay | 13 | Banana | *Musaceae* | *Musa species* | Crop |
| 9 | Chikwawa, Phalombe, Mulanje and Thyolo | 12 | Banana | *Musaceae* | *Musa species* | Crop |
| 11 | Mulanje | 12 | Banana | *Musaceae* | *Musa species* | Crop |
| 12 | Thyolo | 12 | Banana | *Musaceae* | *Musa species* | Crop |
| 13 | Phalombe, Mulanje and Thyolo | 12 | Banana | *Musaceae* | *Musa species* | Crop |
| 14 | Phalombe | 13 | Banana | *Musaceae* | *Musa species* | Crop |

| | | | | | | |
|---|---|---|---|---|---|---|
| 15 | Nsanje | 13 | Banana | *Musaceae* | *Musa species* | Crop |
| 16 | Chikwawa | 12 | Banana | *Musaceae* | *Musa species* | Crop |
| 17 | Zomba | 12 | Banana | *Musaceae* | *Musa species* | Crop |
| 18 | Chikwawa, Nsanje and Zomba | 10 | Banana | *Musaceae* | *Musa species* | Crop |
| 19 | Dedza, Ntcheu, Machinga, Mangochi and Zomba | 9 | Banana | *Musaceae* | *Musa species* | Crop |
| 20 | Dedza | 12 | Banana | *Musaceae* | *Musa species* | Crop |
| 21 | Salima | 13 | Banana | *Musaceae* | *Musa species* | Crop |
| 22 | Machinga and Mangochi | 12 | Banana | *Musaceae* | *Musa species* | Crop |
| 23 | Lilongwe | 12 | Banana | *Musaceae* | *Musa species* | Crop |
| 24 | Dedza, Lilongwe, Machinga | 7 | Banana | *Musaceae* | *Musa species* | Crop |
| 25 | Chitipa and Karonga | 1 | Custard apple | *Annonaceae* | *Annona squamosa* | Crop |
| | | 2 | Mango | *Anacardiaceae* | *Mangifera indica* | Crop |
| | | 3 | Beans | *Fabaceae* | *Phaseolus vulgaris* | Crop |
| | | 4 | Beans | *Fabaceae* | *Phaseolus vulgaris* | Crop |
| | | 5 | Coffee | *Rubiaceae* | *Coffea arabica* | Crop |
| | | 6 | Citrus lemon | *Rutaceae* | *Citrus limon* | Crop |
| | | 7 | Garden pea | *Fabaceae* | *Pisum sativum* | Crop |
| | | 8 | Cacao | *Malvaceae* | *Theobroma cacao* | Crop |
| | | 9 | Cacao | *Malvaceae* | *Theobroma cacao* | Crop |
| | | 10 | Tomato | *Solanaceae* | *Solanum lycopersicum* | Crop |

| | | | | | | |
|---|---|---|---|---|---|---|
| | | 11 | Monkey bread | *Fabaceae* | *Piliostigma thonningii* | Weed (tree) |
| | | 12 | Trichilia (Ndilolo) | *Meliaceae* | *Trichilia emetica* | weed |
| | | 13 | Faba beans | *Fabaceae* | *Vicia faba* | Crop |
| | | 14 | Mulberry | *Moraceae* | *Morus rubra* | Crop |
| | | 15 | Cassava | *Euphorbiaceae* | *Manihot esculenta* | Crop |
| 26 | Nkhatabay and Rumphi | 1 | Amaranthus | *Amaranthaceae* | *Amaranthus retroflexus* | weed |
| | | 2 | Ginger | *Zingiberaceae* | *Zingiber officinale* | weed |
| | | *3* | *Bothriocline longipes* | *Asteraceae* | *Bothriocline longipes* | Weed |
| | | 4 | Pigeon pea | *Fabaceae* | *Cajanus cajan* | Crop |
| | | *5* | Eagle fern | *Dennstaedtiaceae* | *Pteridium aquilinum* | Weed |
| | | 6 | Tangerine | *Rutaceae.* | *Citrus reticulanta* | Crop |
| | | 7 | Marigold | *Asteraceae* | *Tithonia diversifolia* | Weed |
| | | 8 | Sweet potato | *Convolvulaceae* | *Ipomoea batatas* | Crop |
| | | 9 | Pineapple | *Bromeliaceae* | *Ananas comosus* | Crop |
| | | 10 | Cassava | *Euphorbiaceae* | *Manihot esculenta* | Crop |
| | | 11 | Cassava | *Euphorbiaceae* | *Manihot esculenta* | Crop |
| | | 12 | Cassava | *Euphorbiaceae* | *Manihot esculenta* | Crop |
| | | 13 | Cassava | *Euphorbiaceae* | *Manihot esculenta* | Crop |
| | | 14 | Cassava | *Euphorbiaceae* | *Manihot esculenta* | Crop |
| | | 15 | Cassava | *Euphorbiaceae* | *Manihot esculenta* | Crop |
| 27 | Lilongwe and Nkhotakota | 1 | Pepper | *Solanaceae* | *Capsicum frutescens* | Crop |
| | | 2 | Sugarcane | *Andropogoneae* | *Saccharum officinarum* | Crop |
| | | 3 | Sweet potato | *Convolvulaceae* | *Ipomoea batatas* | Crop |
| | | 4 | Sugarcane | *Andropogoneae* | *Saccharum officinarum* | Crop |
| | | 5 | Jimson plant | *Solanaceae* | *Datura stramonium* | weed |
| | | 6 | Okra | *Malvaceae* | *Abelmoschus esculentus* | Crop |
| | | 7 | Sugarcane | *Andropogoneae* | *Saccharum officinarum* | Crop |
| | | 8 | Desmodia silverleaf | *Fabaceae* | *Desmodium uncinatum* | Weed |

| | | | | | | |
|---|---|---|---|---|---|---|
| | | 9 | Sugarcane | *Andropogoneae* | *Saccharum officinarum* | Crop |
| | | 10 | Cassava | *Euphorbiaceae* | *Manihot esculenta* | Crop |
| 28 | Dedza, Ntcheu and Salima | 1 | Castor oil | *Euphorbiaceae* | *Ricinus communis* | Weed |
| | | 2 | Tangerine | *Rutaceae.* | *Citrus reticulanta* | Crop |
| | | 3 | Mulberry | *Moraceae* | *Morus rubra* | Crop |
| | | 4 | Pawpaw | *Caricaceae* | *Carica papaya* | Crop |
| | | 5 | Peach | *Rosaceae* | *Prunus persica* | Crop |
| | | 6 | Guava | *Myrtaceae* | *Psidium guajava* | Crop |
| | | 7 | Castor oil | *Euphorbiaceae* | *Ricinus communis* | weed |
| | | 8 | Bitter orange | *Rutaceae* | *Citrus aurantium* | Crop |
| | | 9 | Pawpaw | *Caricaceae* | *Carica papaya* | Crop |
| | | 10 | Pumpkin | *Cucurbitaceae* | *Cucurbita argyrosperma* | Crop |
| | | 11 | Zimati weed | *Amaranthaceae* | *Cyathula uncinulata* | weed |
| | | 12 | Pepper | *Solanaceae* | *Capsicum frutescens* | Crop |
| 29 | Mulanje and Thyolo | 1 | Sugarcane | *Andropogoneae* | *Saccharum officinarum* | Crop |
| | | 2 | African egg plants | *Solanaceae* | *Solanum aethiopicum* | Weed |
| | | 3 | Taro | *Araceae* | *Colocasia esculenta* | Crop |
| | | 4 | Sorghum | *Poaceae* | *Sorghum bicolor* | Crop |
| | | 5 | Pineapple | *Bromeliaceae* | *Ananas comosus* | Crop |
| | | 6 | Peach | *Rosaceae* | *Prunus persica* | Crop |
| | | 7 | Pigeon pea | *Fabaceae* | *Cajanus cajan* | Crop |
| | | 8 | Woolly lion's ear | *Labiatae* | *Leonotis mollissima* | Weed |
| | | 9 | Taro | *Araceae* | *Colocasia esculenta* | Crop |
| | | 10 | Taro | *Araceae* | *Colocasia esculenta* | Crop |
| | | 11 | Taro | *Araceae* | *Colocasia esculenta* | Crop |
| | | 12 | Taro | *Araceae* | *Colocasia esculenta* | Crop |
| | | 13 | Gonthi weed | *Araceae* | *Alocasia macrorrhiza* | Weed |
| | | 14 | African arrowroot | *Cannaceae* | *Canna indica* | Weed |

| | | | | | | |
|---|---|---|---|---|---|---|
| | | 15 | Cassava | *Euphorbiaceae* | *Manihot esculenta* | Crop |
| 30 | Chikwawa and Nsanje | 1 | Tomato | *Solanaceae* | *Solanum lycopersicum* | Crop |
| | | 2 | Taro | *Araceae* | *Colocasia esculenta* | Crop |
| | | 3 | Taro | *Araceae* | *Colocasia esculenta* | Crop |
| | | 4 | Tomato | *Solanaceae* | *Solanum lycopersicum* | Crop |
| | | 5 | Pepper | *Solanaceae* | *Capsicum frutescens* | Crop |
| | | 6 | Pawpaw | *Caricaceae* | *Carica papaya* | Crop |
| | | 7 | Nidorella | *Asteraceae* | *Nidorella auriculata* | Weed |
| | | 8 | Cowpea | *Fabaceae* | *Vigna unguiculata* | Crop |
| | | 9 | Tobacco | *Solanaceae* | *Nicotiana tabacum* | Crop |
| | | 10 | Taro | *Araceae* | *Colocasia esculenta* | Crop |
| | | 11 | Sweet potato | *Convolvulaceae* | *Ipomoea batatas* | Crop |
| | | 12 | Taro | *Araceae* | *Colocasia esculenta* | Crop |
| | | 13 | Taro | *Araceae* | *Colocasia esculenta* | Crop |
| | | 14 | Maize | *Poaceae* | *Zea mays* | Crop |
| | | 15 | Jatropha | *Euphorbiaceae* | *Jatropha curcas* | Weed |
| 31 | Phalombe, Mangochi and Zomba | 1 | Custard apple | *Annonaceae* | *Annona squamosa* | Crop |
| | | 2 | Maize | *Poaceae* | *Zea mays* | Crop |
| | | 3 | Lemon grass | *Poaceae* | *Cymbopogon citratus* | Crop |
| | | 4 | Sugarcane | *Andropogoneae* | *Saccharum officinarum* | Crop |
| | | 5 | Pawpaw | *Caricaceae* | *Carica papaya* | Crop |
| | | 6 | Tomato | *Solanaceae* | *Solanum lycopersicum* | Crop |
| | | 7 | Sweet potato | *Convolvulaceae* | *Ipomoea batatas* | Crop |
| | | 8 | Cowpea | *Fabaceae* | *Vigna unguiculata* | Crop |
| | | 9 | Cassava | *Euphorbiaceae* | *Manihot esculenta* | Crop |
