# Supplementary material for: Virome Survey of Banana Plantations and Surrounding Plants in Malawi
Source: Viruses. 2025 Jul 31;17(8):1068. doi: 10.3390/v17081068 (PMC12390665; doi:10.3390/v17081068)
Supplement: Supplementary file 1 [file viruses-17-01068-s001.zip › Table S3. High throughput sequencing pools, host plants and their symptomatic status.pdf]

Table S3. High throughput sequencing pools, host plants and their symptomatic status. This table has names of pooled plants, their symptomatic status, region of collection, RNA ID, cDNA ID, Mid ID, sequencing ID and library numbers. EC1 = External control 1, EC2 = External control 2, NC = Negative control.

Pool #	Original host	Symptomatic status	REGION	RNA ID	cDNA ID	MID ID	Sequencing ID	Library	Fasta Q File
1	Banana	Symptomatic	Chitipa and Karonga	VP1	JM_RT-01	1	J1-01	Library 1	1MDJ-LDF 001 sametag R1 & R2
2	Banana	Asymptomatic	Chitipa	VP2	JM_RT-02	4	J1-04		1MDJ-LDF 004 sametag R1 & R2
3	Banana	Asymptomatic	Chitipa	VP3	JM_RT-03	7	J1-07		1MDJ-LDF 007 sametag R1 & R2
4	Banana	Asymptomatic	Karonga	VP4	JM_RT-04	11	J1-11		1MDJ-LDF 011 sametag R1 & R2
5	Banana	Symptomatic	Nkhatabay, Nkhotakota and Rumphi	VP5	JM_RT-05	13	J1-13		1MDJ-LDF 013 sametag R1 & R2
6	Banana	Asymptomatic	Nkhatabay	VP6	JM_RT-06	15	J1-15		1MDJ-LDF 015 sametag R1 & R2
8	Banana	Asymptomatic	Nkhatabay	VP7	JM_RT-07	16	J1-16		1MDJ-LDF 016 sametag R1 & R2
9	Banana	Symptomatic	Chikwawa, Phalombe, Mulanje and Thyolo	VP8	JM_RT-08	18	J1-18		1MDJ-LDF 018 sametag R1 & R2
10	Banana	Asymptomatic	Mulanje	VP9	JM_RT-09	93	J1-93		1MDJ-LDF 093 sametag R1 & R2
EC1	Orchid	Symptomatic	Gembloux, Belgium	VP10	JM_RT-10	84	J1-84		1MDJ-LDF 084 sametag R1 & R2
11	Banana	Asymptomatic	Thyolo	VP11	JM_RT-11	78	J1-78		1MDJ-LDF 078 sametag R1 & R2
EC2	Tomato	Symptomatic	Gembloux, Belgium	VP12	JM_RT-12	95	J1-95		1MDJ-LDF 095 sametag R1 & R2

7	Banana	Symptomatic	Nkhotakota	VP13	JM_RT-13	1	J2-01	Libray 2	2MDJ-LDF 001 sametag R1 & R2
12	Banana	Asymptomatic	Phalombe, Mulanje and Thyolo	VP14	JM_RT-14	4	J2-04		2MDJ-LDF 004 sametag R1 & R2
13	Banana	Asymptomatic	Phalombe	VP15	JM_RT-15	7	J2-07		2MDJ-LDF 007 sametag R1 & R2
14	Banana	Asymptomatic	Nsanje	VP16	JM_RT-16	11	J2-11		2MDJ-LDF 011 sametag R1 & R2
15	Banana	Asymptomatic	Chikwawa	VP17	JM_RT-17	13	J2-13		2MDJ-LDF 013 sametag R1 & R2
16	Banana	Asymptomatic	Zomba	VP18	JM_RT-18	15	J2-15		2MDJ-LDF 015 sametag R1 & R2
17	Banana	Symptomatic	Chikwawa, Nsanje and Zomba	VP19	JM_RT-19	16	J2-16		2MDJ-LDF 016 sametag R1 & R2
18	Banana	Asymptomatic	Dedza, Machinga, Mangochi, Ntcheu and Zomba	VP20	JM_RT-20	18	J2-18		2MDJ-LDF 018 sametag R1 & R2
19	Banana	Asymptomatic	Dedza	VP22	JM_RT-22	93	J2-93		2MDJ-LDF 093 sametag R1 & R2
NC	PCR H_2O		Gembloux, Belgium	VP23	JM_RT-23	84	J2-84		2MDJ-LDF 084 sametag R1 & R2
20	Banana	Symptomatic	Mangochi	VP21	JM_RT-21	78	J2-78		2MDJ-LDF 078 sametag R1 & R2
EC2	Tomato	Symptomatic	Gembloux, Belgium	VP24	JM_RT-24	95	J2-95		2MDJ-LDF 095 sametag R1 & R2
21	Banana	Asymptomatic	Salima	VP28	JM_RT-28	11	J3-11	Libray 3	3MDJ-LDF 011 sametag R1 & R2

22	Banana	Symptomatic	Machinga and Mangochi	VP25	JM_RT-25	1	J3-01		3MDJ-LDF 001 sametag R1 & R2
23	Banana	Asymptomatic	Lilongwe	VP26	JM_RT-26	4	J3-04		3MDJ-LDF 004 sametag R1 & R2
24	Banana	Asymptomatic	Dedza, Lilongwe and Machinga	VP27	JM_RT-27	7	J3-07		3MDJ-LDF 007 sametag R1 & R2
25	Mulburry, Beans, Ndilola (weed), Custard apple, Coffee, *Piliostigma thonningii*, citrus lemon, Garden pea, Tomato, *Vicia faba*, Cacao and cassava .	Mixed	Chitipa and Karonga	VP29	JM_RT-29	13	J3-13		3MDJ-LDF 013 sametag R1 & R2
26	*Pteridium aquilinum*, Sweet potato, *Tithonia diversifolia,* (Marigold)*,* *Bothriocline longipe*, Pine apple, Pigeon pea, Tangerines, ginger and cassava	Mixed	Nkhatabay and Rumphi	VP30	JM_RT-30	15	J3-15		3MDJ-LDF 015 sametag R1 & R2

27	Sugarcane, Okrah, Pepper, Sweet potato, *Datura stramonium*, Desmodium silverleaf, cassava and Amaranthus	Mixed	Lilongwe and Nkhotakota	VP31	JM_RT-31	16	J3_16		3MDJ-LDF 016 sametag R1 & R2
28	Castor oil, Pawpaw, Guava, *Cyathula uncinulata*, Pepper, Pumpkin, Bitter orange, Tangerines, Peach and Mulberry	Mixed	Dedza, Ntcheu and Salima	VP32	JM_RT-32	18	J3_18		3MDJ-LDF 018 sametag R1 & R2
29	Sugarcane, Taro, Pineapple, Sorghum, Pigeon pea, *Alocasia macrorrhiza*, maize, *Canna indica*, *Abelmoschus esculentus,* cassava, Peaches and *Leonotis mollissima*	Mixed	Mulanje and Thyolo	VP34	JM_RT-34	78	J3_78		3MDJ-LDF 078 sametag R1 & R2

30	Taro, cowpea, Jatropha, Pepper, Tomato, Tobacco, Maize, Pawpaw, sweet potato and *Nidorella auriculata*	Mixed	Chikwawa and Nsanje	VP35	JM_RT-35	84	J3_84		3MDJ-LDF 084 sametag R1 & R2
31	Custad fruit, Cassava, Tomato, Maize, cowpea, Sugarcane, Sweet potato, Lemon grass and Pawpaw	Mixed	Phalombe, Mangochi and Zomba	VP33	JM_RT-33	93	J3_93		3MDJ-LDF 093 sametag R1 & R2
