# Supplementary material for: Virome Survey of Banana Plantations and Surrounding Plants in Malawi
Source: Viruses. 2025 Jul 31;17(8):1068. doi: 10.3390/v17081068 (PMC12390665; doi:10.3390/v17081068)
Supplement: Supplementary file 1 [file viruses-17-01068-s001.zip › Table S4. Tymoviridae and Totiviridae reference genomes from NCBI.pdf]

Table S4. Tymoviridae and Totiviridae reference genomes from NCBI. This table has the Tymovirids and Totivirids reference accessons names and number from the NCBI genbank the used in the construction of phylogenetic trees.

**Tymovirids reference accessions**	**Accession number**		**Totivirids reference accessions**	**Accession number**
Alcea yellow mosaic virus	OP227146		Alternaria arborescens victorivirus 1	NC 040793
Alfalfa virus F	NC 040565		Armigeres subalbatus virus	NC 014609
Anagyris vein yellowing virus	NC 011559		Aspergillus foetidus slow virus 1	NC 038928
Andean potato latent virus	NC 020470		Beauveria bassiana victorivirus	NC 024151
Andean potato mild mosaic virus	NC 020471		Birch toti-like virus	PP740463
Asclepias asymptomatic virus	NC 015523		Black raspberry virus F	NC 009890
Bee Macula-Like virus 2	NC 040556		Bursera graveolens associated totivirus 1	ON988291
Belladonna mottle virus	OR082757		Camponotus yamaokai virus	NC 027212
Blackberry virus S	NC 038328		Carrot-associated toti-like virus	OP886480
Bombyx mori latent virus	NC 038331		Chalara elegans RNA Virus 1	NC 005883
Bombyx mori Macula-like virus	NC 015524		Culex tritaeniorhynchus totivirus	NC 040670
Camellia-associated marafivirus	MT036048		Diatom colony associated dsRNA virus 12	NC 040775
Chayote mosaic virus	NC 002588		Drosophila melanogaster totivirus	NC 013499
Chiltepin yellow mosaic virus	NC 014127		Eimeria brunetti RNA virus 1	NC 002701
Citrus sudden death-associated virus	NC 006950		Erysiphales associated totivirus 18	MN628289
Culex originated Tymoviridae-like virus	NC 018703		Geotrichum candidum totivirus 2	ON862168
Diascia yellow mottle virus	NC 011086		Giardia lamblia virus	NC 003555
Dulcamara mottle virus	NC 007609		Golden shiner totivirus	NC 030295
Eggplant mosaic virus	NC 001480		Leishmania RNA virus 1 – 1	NC 002063
Erysimum latent virus	NC 001977		Leptopilina boulardi Toti-like virus	NC 025218
Erysimum leaf mottle virus	ON398515		Loquat associated totivirus 1	OK318989
Fig fleck-associated virus	NC 015229		Maize associated totivirus	MK066243
Grapevine asteroid mosaic associated virus	NC 031692		Nigrospora oryzae victorivirus 1	NC 030224
Grapevine fleck virus	NC 003347		Panax notoginseng virus A	NC 029096
Grapevine Red Globe virus	NC 030693		Papaya meleira virus	NC 028378

Grapevine rupestris vein feathering virus	NC 034205	Peach-associated virus 2	MN905504
Grapevine Syrah virus 1	NC 012484	Piscine myocarditis virus AL V-708	NC 015639
Kennedya yellow mosaic virus	NC 001746	Puccinia striiformis totivirus 5	KY207365
Maculavirus vitis	OR701334	Rosellinia necatrix victorivirus 1	NC 021565
Maize rayado fino virus	NC 002786	Saccharomyces kudriavzevii virus L-A1	NC 032106
Marafivirus pruni	MH898957	Sanya totivirus 6	MZ209873
Marafivirus syrahense	OR787586	Scheffersomyces segobiensis virus L	NC 038697
Naranjilla chlorotic mosaic virus	NC 077008	Sogatella furcifera totivirus 2	NC 040704
Naranjilla mild mosaic virus	NC 077017	Taro-associated totivirus L	MN119621
Nectarine marafivirus M	NC 029063	Tea-oil camellia-associated totivirus 1	MW025466
Nemesia ring necrosis virus	NC 011538	Tuber aestivum virus 1	NC 038698
Oat blue dwarf virus	NC 001793	Ustilaginoidea virens RNA virus 5	NC 028477
Okra mosaic virus	NC 009532	Vaccinium-associated virus C	OR475296
Olive latent virus 3	NC 013920		
Ononis yellow mosaic virus	NC 001513		
Passion fruit yellow mosaic virus	MW393829		
Peach chlorotic mottle virus	NC 009892		
Peach virus D	NC 033828		
Pennisetum glaucum marafivirus	MZ305310		
Physalis mottle virus	NC 003634		
Plantago mottle virus	NC 011539		
Poinsettia mosaic virus	NC 002164		
Ranunculus virus M	PQ179277		
Sea buckthorn marafivirus	ON149451		
Sorghum almum marafivirus	NC 077048		
Styphnolobium tymo-like virus	OR934935		
Switchgrass mosaic virus	NC 015522		
Tomato blistering mosaic virus	NC 021851		
Triticum aestivum marafivirus	OR162022		
Turnip yellow mosaic virus	NC 004063		

Valeriana jatamansi tymovirus 1	OQ730267			
Varroa Tymo-like virus	NC 027619			
