# Supplementary material for: Virome Survey of Banana Plantations and Surrounding Plants in Malawi
Source: Viruses. 2025 Jul 31;17(8):1068. doi: 10.3390/v17081068 (PMC12390665; doi:10.3390/v17081068)
Supplement: Supplementary file 1 [file viruses-17-01068-s001.zip › Table S5. viruses tested by RT-PCR on plants and their primers.pdf]

Table S5. Listing of the viruses tested by RT-PCR on the non-banana plants and the primers used. This table has virus names, their primers with their names and sequences, annelling temperatures and amplicon size

Virus name	Primer name	Primer sequence	Anneling temp. (ºC)	Amplicon size (pb)
Pea seed-borne virus	PSbMV- F<br>PSbMV-R	GAGAAGTGAGAGGAGCGGATC<br>GCTGAGATCTTTTCAAGCCTTGG	60	173
Ginger tymo-like virus (Novel)	TymNV1252-1274F<br>TymNV G2061-2039R	GAGGCTCTGCTCTTGCCC<br>CTGAAGTAGTGGTCGTGCGA	60	809
Sweet potato leaf curl virus	SpLCV F<br>SpLCV R	CTAGGATGACAGGGCGAATTC<br>AGATCTATTATTGTGCGAATCATAGAAA	58	773
Citrus tristeza virus	Citrus triste v15624-15645F<br>Citrus triste v15923-15903R	GTGCACTATATCCACGAGTACT<br>CCTGGAAGAGAACCCGTAAGA	57	258
Pepper derived totivirus (Novel)	TotiRcFG4466-4487F<br>TotiRcFG5134-5114R	AAGTGTAATGTGCTGTCGGTAG<br>GGCTTTGTCCACAAATGGTAC	63	750
Pepper vein yellow virus	PVYV1-F<br>PVYV1-R	TCGTTTGTCTCGCGTTGCTTTTCG<br>ATCGTTGGAACCCTGGGATCTCTT	62	1.1 kb
Potato virus Y	PVY-F<br>PVY-R	ATACTCGRGCAACTCAATCACA<br>CCATCCATCATAACCCAAACTC	64	166
Chickpea chlorotic dwarf virus	CpCDV-F<br>CpCDV-R	GGACGTGGACTGTGTCTAGG<br>TGGGTATTTCCGAGTGCAGC	59	300
Tomato mosaic virus	ToMV-F<br>ToMV-R	CTCGTCGGCAGGTATATCGT<br>GGCACCAAACATGTCCCAAA	59	174
