# Supplementary material for: Virome Survey of Banana Plantations and Surrounding Plants in Malawi
Source: Viruses. 2025 Jul 31;17(8):1068. doi: 10.3390/v17081068 (PMC12390665; doi:10.3390/v17081068)
Supplement: Supplementary file 1 [file viruses-17-01068-s001.zip › Table S7. Virus name, its family and genus names, their associated host plants and the collected sites.pdf]

Table S7: Virus name, its family and genus names, their associated host plants and the collected sites. * : Not confirmed by (RT-)PCR from individual samples. ** : host plant confirmation by (RT-)PCR from individual samples. First report indicates the first report from Malawi. Purple boxes = new virus host plants. On status column, brown boxes = confirmed by RT-PCR and grey boxes = not confirmed by RT-PCR.

Virus name	Family	Genus	Genome	Host Plant **	Collected site	Status	Accession number
**Ginger tymo-like virus**	*Tymoviridae*	*Maculavirus*	(+) ssRNA	*Zingiber officinale*	Nkhatabay	New species	PQ682664 PV831853-54
**Pepper derived totivirus**	*Totiviridae*	*Totivirus*	dsRNA	*Capsicum frutescens*	Lilongwe	New species	PQ682663 PV930374-76
**Chickpea chlorotic dwarf virus**	Geminiviridae	*Mastrevirus*	ssDNA	*Vigna unguiculata*	Nsanje	First report	PV829524
**Pea seed-borne mosaic virus**	*Potyviridae*	*Potyvirus*	(+) ssRNA	*Piusm sativum*	Chitipa	First report	PV829525
**Pepper vein yellows virus**	*Luteoviridae*	*Polerovirus*	(+) ssRNA	*Capsicum frutescens*	Lilongwe and Nsanje	First report	PV463389-90
**Sweet potato leaf curl virus**	Geminiviridae	Begomovirus	ssDNA	*Ipomea batatas*	Rumphi and Nkhotakota	First report	PV463387-88
**Citrus tristeza virus**	*Closteroviridae*	*Closterovirus*	(+) ssRNA	*Citrus paradisi* & *Citrus reticulata*	Dedza<br>Dedza and Nkhatabay	First report	PV463392 & PV492511
**Potato virus Y**	*Potyviridae*	*Potyvirus*	(+) ssRNA	*Ipomea batatas*	Rumphi	First report	PV463391
**Tomato mosaic virus**	*Virgaviridae*	*Tobamovirus*	(+) sRNA	*Solanum lycopersicum*	Phalombe	First report	PV492510

Virus name	Family	Genus	Genome	Host Plant *	Collected site	Status	Accession number
**Bean yellow dwarf virus**	*Geminiviridae*	*Mastrevirus*	ssDNA	*Vigna unguiculata* L. Walp	Chikwawa	Reported	N/A
**Banana mild mosaic virus**	Betaflexiviridae	Banmivirus	(+) ssRNA	Banana (*Musa* spp)	Widespread	Reported	N/A
**Banana streak OL virus**	Caulimoviridae	Badnavirus	dsDNA	Banana (*Musa* spp)	Widespread	Reported	N/A
**Banana bunchy top virus**	*Nanoviridae*	*Babuvirus.*	ssDNA	Banana (*Musa* spp)	All banana cultivation zones	Already Report	
**Manihot esculenta-associated virus 1**	*Closteroviridae*	*Ampelovirus*	(+) ssRNA	*Manihot esculenta*	Thyolo Nkhatabay & Nkhotakota	Already reported	N/A
**Maize streak virus**	*Geminiviridae*	*Mastrevirus*	ssDNA	*Zea mays*	Phalombe	Already Reported	N/A
**East Africa cassava mosaic virus**	*Geminiviridae*	*Begomovirus*	ssDNA	*Manihot esculenta*	Mulanje, Thyolo, Rumphi, Nkhatabay & Karonga	Already Reported	N/A
**Cassava streak virus**	*Potyviridae*	*Ipomovirus*	(+) ssRNA	*Manihot esculenta*	Thyolo, Phalombe, Kalonga, Nkhotakota, Nkhatabay and Rumphi	Already Reported	N/A
**Ethiopian tobacco bushy top virus**	*Tombusviridae*	*Umbravirus*	(+) ssRNA	*Nicotiana tabacum*)	Chikwawa	Already Reported	N/A

Virus name	Family	Genus	Genome	Host Plant *	Collected site	Status	Accession number
**Pod pepper vein yellow virus**	*Solemoviridae*	*Polerovirus*	(+) ssRNA	*Capsicum frutescens)*	Nsanje	Reported	N/A
**Tobacco mosaic virus**	*Virgaviridae*	*Tobamovirus*	(+) ssRNA	*Solanum lycopersicum*	Phalombe	Reported	N/A
**Peanut mottle virus**	*Potyviridae*	*Potyvirus*	(+) ssRNA	*Cajanus cajan*	Thyolo	Already Reported	N/A
**Arhar cryptic virus**	*Partitiviridae*	*Deltapartitivirus*	dsRNA	Test not done	Not confirmed by RT-PCR	First report	PV829522
**Sugarcane bacilliform virus**	*Caulimoviridae*	*Badnavirus*	dsDNA	Test not done	Not confirmed by RT-PCR	First report	PV829523
