# Supplementary material for: Virome Survey of Banana Plantations and Surrounding Plants in Malawi
Source: Viruses. 2025 Jul 31;17(8):1068. doi: 10.3390/v17081068 (PMC12390665; doi:10.3390/v17081068)
Supplement: Supplementary file 1 [file viruses-17-01068-s001.zip › Table S8A. Nucleotide mismatches analysis between CP2 primer and BanMMV consensus sequence.pdf]

Table S8A. Nucleotide mismatches analysis between CP2 primer and BanMMV consensus sequence. This table shows the analysis of nucleotide mismatches between CP2 primer and the consensus banana mild mosaic virus sequences of the samples. Letters indicate the observed mismatches. Red colour in the RT-PCR column means BanMMV detection

CP2 primer	5´	BanMMV CP2 Primer analysis																						3´	Coverage	RT-PCR
	T	G	C	C	A	A	C	T	G	A	Y	G	A	R	G	A	G	C	T	R	A	A	T	G	C		
J1-01					T			A																		3X	
J1-04					G	C		G			T															13X	
J1-07						G		C	T		C			G	A	G	T			C	C		G		A	3x	
J1-11																										n.a	
J1-13					T			A																		28x	
J1-15					T			A																		21x	
J1-16		A			T			A			T						A									3x	
J1-18					T			A									A									8x	
J1-78					T			A									A									20x	
J1-93					T			A									A									3x	
J2-01					T			A																		8x	
J2-04					T			A									A									20x	
J2-07					T			A									A									12x	
J2-11																										n.a	
J2-13					T			A									A									42x	
J2-15					T			A									A									13x	
J2-16																										n.a	
J02-18					T			A																		7x	
J02-78					T			A									A									19x	
J02-93																										n.a	
J03-01																										<3x	
J03-04					T			A																		5x	
J03-07					C			G									A									3x	
J03-11					T			A																	G	7x	
