# Supplementary material for: Virome Survey of Banana Plantations and Surrounding Plants in Malawi
Source: Viruses. 2025 Jul 31;17(8):1068. doi: 10.3390/v17081068 (PMC12390665; doi:10.3390/v17081068)
Supplement: Supplementary file 1 [file viruses-17-01068-s001.zip › Table S8B. In depth mismatch analysis of banana mild mosaic virus sequences and BanMMV CP 9 primers.pdf]

Table S8B. In depth mismatch analysis of banana mild mosaic virus sequences and BanMMV CP 9 primers. This table shows the analysis of nucleotide mismatches between CP9 primer and the consensus banana mild mosaic virus sequences of the samples. Letters indicate the observed mismatches. Red colour in the RT-PCR column means BanMMV detection

CP9 Seq.	5´	BanMMV CP9 Primer analysis																	3´	Coverage	RT-PCR
	T	C	T	G	A	T	K	C	A	G	C	A	A	T	H	A	T	S	C	C		
J01-01			A			C			G			T									1x	Negative
J01-04			A			C						T									9x	Negative
J01-07			A									T									3x	Negative
J01-11																					n.a	Negative
J01-13			A			C						T									28x	Negative
J01-15			A			C						T									18x	Negative
J01-16			A			C															2x	Negative
J01-18			A			C						T									8x	Negative
J01-78			A			C						T									12x	Negative
J01-93			A			C						T									3x	Positive
J02-01			A			C						T									8x	Negative
J02-04			A			C						T									21x	Positive
J02-07			A			C						T									10x	Positive
J02-11																					n.a	Negative
J02-13			A			C						T									41x	Negative
J02-15			A			C						T									13x	Negative
J02-16																					n.a	Negative
J02-18			A			C						T									7x	Negative
J02-78			A			C						T									19x	Negative
J02-93																					n.a	Negative
J03-01			A			C						T									2x	Negative
J03-04			A			C						T									6x	Negative
J03-07			A			C						T									4x	Negative
J03-11			A			C						T									6x	Negative
